# Oak Biomass in the Form of Wood, Bark, Brushwood, Leaves and Acorns in the Production Process of Multifunctional Biochar

**DOI:** 10.3390/molecules27217191

**Published:** 2022-10-24

**Authors:** Bogdan Saletnik, Aneta Saletnik, Grzegorz Zaguła, Marcin Bajcar, Czesław Puchalski

**Affiliations:** Department of Bioenergetics, Food Analysis and Microbiology, Institute of Food Technology and Nutrition, College of Life Sciences, University of Rzeszow, 2D Ćwiklińskiej Street, 35-601 Rzeszow, Poland

**Keywords:** multifunctional biochar, pyrolysis, oak biomass, explosibility, macro- and micronutrients

## Abstract

Biochar from forest biomass and its remains has become an essential material for environmental engineering, and is used in the environment to restore or improve soil function and its fertility, where it changes the chemical, physical and biological processes. The article presents the research results on the opportunity to use the pyrolysis process to receive multifunctional biochar materials from oak biomass. It was found that biochars obtained from oak biomass at 450 and 500 °C for 10 min were rich in macronutrients. The greatest variety of the examined elements was characterized by oak-leaf pyrolysate, and high levels of Ca, Fe, K, Mg, P, S, Na were noticed. Pyrolysates from acorns were high in Fe, K, P and S. Oak bark biochars were rich in Ca, Fe, S and contained nitrogen. In addition, biomass pyrolysis has been found to improve energy parameters and does not increase the dust explosion hazard class. The oak biomass pyrolytic at 450 and 500 °C after 10 min increases its caloric content for all samples tested by at least 50%. The highest caloric value among the raw biomass tested was observed in oak bark: 19.93 MJ kg^−1^ and oak branches: 19.23 MJ kg^−1^. The mean and highest recorded *K_st_*_max_ were 94.75 and 94.85 bar s^−1^, respectively. It can be concluded that pyrolysis has the potential to add value to regionally available oak biomass. The results described in this work provide a basis for subsequent, detailed research to obtain desired knowledge about the selection of the composition, purpose, and safety rules of production, storage, transport and use of biochar materials.

## 1. Introduction

As reported by the International Biochar Initiative (IBI), biochar is a fine-grained carbonization product characterized by high organic carbon content and low susceptibility to degradation. It is obtained by pyrolysis of biomass and biodegradable waste [1,2]. Biochar has physical and chemical properties suitable for the safe and long term storage of carbon in our environment. It is produced under certain controlled conditions, which makes the carbon therein more stable and can be converted into utility products [3,4,5,6,7]. The most commonly used biomass is wood and its residues and by-products, i.e., wood chips and sawdust, agricultural residues and their by-products, e.g., quinoa, rice husks, manure, as well as waste from the paper industry, household waste and wastewater [6,7]. Until the 19th century, biomass was mainly used for the production of thermal energy in the process of direct combustion. Combustion by-products such as ashes were used to fertilize soils [8]. The growing demand for energy and increasing awareness of the need to protect the environment have led scientists to look for and exploit new alternative sources of biochar [6]. A promising source of renewable energy, in liquid form, is biofuel from the pyrolysis of microalgae. Advanced development of microbiological technology has favored biofuel production from microalgal biomass as the third generation of bioenergy. In view of compositions, microalgal carbohydrates, lipids and proteins are ideal as feedstock for bio-oil production, as well incite the potential of fossil fuels replacement by microalgal biofuel [9,10]. Popular raw materials for biochar production include rice husk, wood bark, sugar beet waste, empty fruit bunches, dairy fertilizer, pine wood, wood chips, organic waste, plant residues, human manure and poultry manure [11,12]. The use of biomass obtained from waste for the production of biochar is an effective way of converting them into a useful substance of increased value [6,7]. Among alternative sources of biomass, researchers emphasize using residual sources [13]. However, use raw biomass of residual origin can be problematic. Crude waste biomass has a non-uniform structure, a higher humidity and a much lower calorific value [14]. For these reasons, the use of raw biomass is hardly economically viable. The implementation of mechanical, thermal or organic processes can greatly improve the physical properties of the original biomass and increases its profitability [15].

Pyrolysis is one of the main thermal treatment processes for materials [16,17,18]. In the pyrolysis process, biomass is converted into solids with a high degree of carbonization, so called carbonizate bio-oil, otherwise called pyrolysis oil and gas. The pyrolysis process is used to obtain biochar (carbonizate) [19]. This process takes place under anaerobic conditions or with access to a small amount of oxygen, insufficient to burn the raw material [20]. In biochar production, the procedure begins with biomass drying, where the molecule is further heated to release volatile materials from the solid [21]. Pyrolysis usually proceeds at temperatures between 300 and 700 °C, however, pyrolysis as a process can be carried out at higher temperatures. In the pyrolysis process, depending on the parameters used, different biochar content can be obtained, bio-oil and gas. Due to differences in the use of process parameters (process time and heating rate), three types of pyrolysis are distinguished: fast, moderate and slow [21,22]. Fast pyrolysis (temperature 500 °C with peak (final) temperature ultimate for 1 s produces about 12% of biochar, 60% bio-oil and 20% syngas [21]. Temperature is one of the major factors for products distribution in fast pyrolysis process [23,24]. Biomass fast pyrolysis is a promising technology to generate renewable fuel intermediates. However, its commercialization is limited due to the multi-scale challenges in understanding the complex physicochemical phenomena involved in the conversion process. Physics-based multi-scale modeling is used is a tool to investigate these complex multiscale phenomena simultaneously [25,26,27]. By using moderate pyrolysis (temperature 500 °C, ultimate temperature maintained for 10–20 s)—about 20% of biochar can be obtained. The yield of the moderate pyrolysis product is 50% liquid, 20% solid and 30% gaseous products [21]. Some sources say that more than 70% of biomass is turned into bio-oil [28]. The highest content of biochar, at the level of ≥35%, can be obtained by slow pyrolysis (at a temperature of 400–500 °C with the ultimate temperature maintained for 5–30 min) [28,29]. The typical yield of the slow pyrolysis product is 30% liquid, 35% solid and 35% gas [24]. The use of high temperature, above 800 °C, and a short duration of the process at the ultimate temperature (gasification) lead to biochar at the level of 10% and 65% of the biomass becomes gas [28,29]. The low biochar content may also be related to the presence of oxygen and water in the reactor [30]. The main gases produced in the pyrolysis of biomass are a mixture of H_2_, hydrocarbon gases (C1–C4), CO_2_, CO, and H_2_S. The pyrolytic gases can be classified into three categories including incombustible gases (H_2_O and CO_2_), combustible gasses (CO and CH_4_), and N-containing gases (NH_3_ and HCN). A lower pyrolysis temperature results in lower yield of gases, whereas with an increase in temperature, the biomass undergoes further secondary reactions to form pyrolytic gases. As revealed from the literature, the formation of CO_2_ mainly originates from decomposition reactions of carbonyl and carboxyl groups in biomass pyrolysis reaction, whereas the formation of CO mainly results from breaking of C-O-C and C=O bonds. However, H_2_ mainly results from breaking of C-H groups and aromatics. However, CO and CO_2_ are dominant gaseous products at low temperatures and CH_4_ is a dominant product at high temperatures due to lignin depolarization reactions [31]. During the pyrolysis process, formation of polycyclic aromatic hydrocarbons (PAHs) may take place. For lignin, WWA may be generated directly from the aromatic structure of the feedstock. Research is being carried out on the influence of reaction conditions, temperature, heating rate, and reaction atmosphere on the formation of polycyclic aromatic hydrocarbons (PAHs) from lignin. Temperature increase from 500 to 900 °C, most PAHs increased with temperature, except 1-methynaphthalene and 2-methynaphthalene, which decreased slightly when the temperature was increased from 800 to 900 °C. With the increase of the temperature, the percentage of 2-ring PAHs decreased and the percentage of 3- and 4-ring PAHs increased. The increase in the total PAH with the temperature could be fitted by a quadratic function. The PAH generation from slow pyrolysis of lignin was much lower than that from fast pyrolysis. In comparison of the PAH generation in different reaction atmospheres, experiments in N-2 produced the most PAHs, followed by the reaction in air and CO_2_. During the pyrolysis/gasification of lignin, it is suggested that there were two kinds of secondary reactions—dehydroxylation and demethoxylation—and they might occur at the same time. Then, PAHs could be formed from secondary reactions of derivatives of benzene, which increased with the increase of the temperature. Slow pyrolysis generated less PAHs because of the limitation of secondary reactions. With the addition of air or CO_2_, derivatives of benzene and phenol could be oxidized; thus, less PAHs were generated. The literature reports that most of the PAHs were concentrated in bio-oil (>70%), with only a small part remaining in biochar and biogas [32,33].

Carbonizate is produced under strictly defined conditions during the pyrolysis process. This results in it being more stable than biomass and of greater utility importance [4,5]. The ratio of carbonizate to biomass is primarily affected by the type of raw material used [29]. The size of the product obtained by pyrolysis of biomass also depends on the process conditions: temperature and processing time at final temperature [30,34]. Higher carbonizate yield can be obtained from biomass raw materials with higher lignin content and lower hemicellulose content [29,35]. The highest efficiency in the production of carbonizates is achieved when raw materials with a high content of lignin are subjected to free pyrolysis at moderate temperatures [36,37]. Biochar generally consists of carbon and minerals. Its physicochemical characteristics such as porosity, organic and inorganic composition, stability and adsorption capacity of nutrients and water are mainly defined by raw material characteristics and pyrolysis parameters [38,39,40,41,42,43,44,45,46,47,48]. Carbonizates formed in high pyrolysis temperatures (>600 °C) are characterized by high pH, high porosity and higher aromaticity. In contrast, the use of lower temperature pyrolysis with slow heating results in higher charred efficiency and higher volatile and oxygen content. Such conditions of the pyrolysis process provide high electrical conductivity of carbonizates and cation exchange capacity [49,50,51,52,53,54], resulting in higher adsorption capacity and greater potential for stable carbon in soil [55]. The skeleton structure of biochar is mainly carbon and minerals with different pore sizes [56]. Biocarbon micropores are responsible for high absorbency and surface area, mesopores are essential in liquid-solid adsorption processes, and macropores are important for soil structure, hydrology, aeration and root movement. The pattern and pore size of biochar depend on the input materials and process temperature used during its formation [57]. The increase in the pyrolysis temperature of woody sapwood produces a biochar with a greater number of pores. It is caused by the thermal decomposition of lignocellulosic components [58]. Most biochars used for soil amendment are alkaline, however, biochar pH values between 3.1 and 12.0 have been reported in the literature. The pH of the biochar is dependent on the feedstock- and production process. Biochars with low ash content, such as those produced using woody feedstocks, generally have lower pH values than biochars with higher ash content, such as those produced using grass, crop residues or manures. Biochars produced under high temperatures (>400 °C) are likely to have greater pH values than the low temperature (<400 °C) biochars from the same feedstock. The pH of biochar may also change post-production depending on the environmental conditions. For example, incubation studies have demonstrated that biochar pH may increase or decrease post-production due to alkaline mineral dissolution or carbon oxidation, respectively [59]. Biochar with a high pH value would cause a significant rise in soil pH with neutral to basic properties but only a slight increase in soil with acidic pH. The outcome of biochar on the exchangeable cation capacity value of soil displays correlation with the fluctuation of Ca^2+^ present and the rise in pH value. Acidic soils such as peat benefited from an increase in the pH but the rise of pH in neutral soil, as those soils in a temperate climate, inhibit the growth of pH-sensitive microbes [1].

The subject literature and the existing quality standards indicate that biocarbon is a material used for non-energy purposes, in particular for soil applications. It is emphasized that the term “biocarbon” was introduced to distinguish traditional char (charcoal) used for energy purposes from a material that can be safely used as a fertilizer (or soil improver (biochar). It is primarily treated with different requirements for these applications [60]. Biochar can be used as an additive to soils, fodder and silage [61,62]. Biochar is a suitable material for immobilizing and removing contaminants from soil and water. It can be used as a supporting raw material in composting and methane fermentation processes [63,64,65,66,67,68]. Biochar is used as a filter for reducing tar in pyrolysis and gasification processes, and as a fuel during pelletization. It has been proven that biochar can be used as a substrate for hydrogen production [69,70,71]. Biochar from forest biomass and residue thereof has become an essential material for environmental engineering [38]. Increased CO_2_ emissions to the atmosphere in recent years have led to a significant disproportion between natural emissions and carbon sinks [21].The world is currently focusing on the problems of global warming and the uncontrolled increase in global temperature, which may lead to an ecological catastrophe. Various actions are taken to prevent global warming of more than 1.5 °C [15]. One of the solutions to this problem is the use of biochar obtained from various types of biomass. When introduced into the soil, it enables long-term carbon sequestration [21]. The carbon (IV) oxide emission over soil respiration is about 10 times higher compared to that produced from the burning of fossil fuel. Furthermore, it is essential to decrease carbon dioxide contaminants from agricultural soil to moderate climate change [1]. Research shows that only a small fraction of biochar is bioavailable—3%, and the remaining 97% directly contributes to long-term C sequestration in soil [43]. According to the literature, by adding biochar to the soil in the amount of 13.5 t/ha, carbon can be stored there for a minimum of 200 years [21]. Research observed that biomass pyrolysates used for bioenergy production have a value of 100 years of sequestration, which corresponds to 12 tons of avoided carbon dioxide emissions [15]. Biochar may lead to the reduction of nitrous oxide and methane emissions from the soil through biotic and abiotic processes [21]. Numerous international research studies confirm that biochar can reduce greenhouse gas emissions per hectare by around 30% [72].

Biochar reduces drought by increasing soil moisture content, thereby inhibiting soil erosion and nutrient leaching [73,74]. Biochar obtained by pyrolysis is used in the environment to restore or improve soil function and fertility, where it changes chemical, physical and biological processes [75,76,77,78,79]. In recent years, the use of biochar as a product for soil enrichment in order to increase crop productivity has been increasing [38,75] especially on sandy and/or acidic soils [38]. Subject literature reports in large numbers about the positive effect of biochar on the availability of nutrients, which makes it a great prospect as a slow-release fertilizer in the soil. When nutrients from biochar are release (especially the pre-adsorbed nutrients) it is solely influenced by its desorption characteristics. Some of its features may have major effects on nutrient desorption from biochar [1]. Zhang et al. revealed that the rates of desorption of ammonium from hardwood biochar rise from about 19% to 29%, due to a decrease in the pyrolyzed temperatures range from 650 to 450 °C [47]. Considering black soil, the minimum per cent of P desorbed over lower P loads (19 mg L^−1^) rises from 35% to 40% with a rise in biochar application rates ranging between 1 and 11%. Researchers specified that above 66% of the P adsorbed by biochar was release at higher P loadings (105 and 250 mg/L). This shows that the percentage desorption of P may increase by enhancing biochar application rates and P loadings. Furthermore, cacao shell biochar desorbed 1487 mg/kg of PO3^−4^ and corncob biochar desorbed 175 mg kg^−1^ of PO3^−4^ [1]. Micropores in biochar allow sorption of dissolved organic matter and improve the activity of microorganisms, which accelerates the remediation of organic contaminants in soils [79,80,81,82,83]. The existence of biochar has implications for permeability, soil response to water, swelling, shrinkage, its aggregation, and the reaction of soil workability to changes in ambient temperature. It changes the physical nature of the soil, causing an increase in the total area of proper soil, which strongly increases the ventilation and structure of the soil [84,85].

Biochar possesses a range of chemical structures and a heterogeneous elemental composition. This variability is based on the conditions of pyrolysis and the biomass parent material. This variability induces a broad spectrum in the observed rates of reactivity and, correspondingly, the overall chemical and microbial stability [43]. The stability and decomposition of biochar are fundamental to understand its persistence in soil, its contribution to carbon (C) sequestration, and thus its role in the global C cycle. Wang et al. meta-analyzed the biochar decomposition in soil and estimated its mean residence time (MRT). The researchers noted that, the decomposed amount of biochar increased logarithmically with experimental duration, and the decomposition rate decreased with time. The biochar decomposition rate varied significantly with experimental duration, feedstock, pyrolysis temperature, and soil clay content. The MRTs of labile and recalcitrant biochar C pools were estimated to be about 108 days and 556 years with pool sizes of 3% and 97%, respectively [43] The scientists has shown that a reliable predictor of overall stability of biochar in soils might be the O:C molar ratio. This ratio is the net result of all of the multiple parameters during the production, cooling and storage of the biochar. Based on the literature studies, biochar with an O:C molar ratio of less than 0.2 are typically the most stable, possessing an estimated half-life of more than 1000 years; biochar with an O:C ratio of 0.2–0.6 have intermediate half-lives (100–1000 years); and, finally biochar with an O:C ratio of greater than 0.6 possess a half-life in the order of over 100 years. [86]. Several many studies have been carried out over the last few years to assess the global impact of biochar on various agricultural soils. Numerous international research studies confirm that biochar increases yield, root mass and microbial activity builds up soil organic matter and improves water-use efficiency. The highest yield increases using pure biochar can be achieved in acidic tropical soils that are poor in soil organic matter [72]. The biomass feedstock and the operating parameters have to be selected with care to obtain a biochar with the desired properties for use on certain types of soil [87]. Biochar addition to sandy soils strongly stimulated SOM mineralization by 20.8%. This indicates that biochar stimulates microbial activities especially in soils with low fertility [43].

Oak is one of the main species of forest trees [88]; it represents 7.9% of all tree species in Polish coniferous and deciduous forests [89]. Oak biomass residues may be a ubiquitous source of bioenergy and biochar [90]. In the pyrolysis process, the wood material is converted into a product with twice the carbon content. Biochars store rapidly decaying C from plant biomass into a much more durable form. Furthermore, the storage capacity of biochar, as opposed to biomass sequestration, is unlimited [76]. Literature reports that, oak pyrolysates are characterized by high Ca supply. Oak wood biochar can be considered a valuable soil amendment, and its properties can be engineered by setting particular pyrolysis conditions. The environmental properties of biochar that are widely affected by pyrolysis temperature and residence time are contents of ash and fixed carbon; elemental composition CHNO, especially carbon content; aromaticity; surface area; total pore volume; pH; surface acidity; cation exchange capacity; functional groups and their ratios; water holding capacity; and nutrient content [38]. It is therefore appropriate to examine and use oak biomass for the production of biochar intended e.g., as fertilizer material. The wood of the trunk, bark, branches, leaves and acorns have different structure and chemical composition. They differ in the content of lignin, cellulose and hemicellulose, pectins and extracts [91].

To date, the authors in the current review of the literature have not found scientific work which compares with each other the thermal and physicochemical properties of biochars obtained in the pyrolysis process from all types of oak biomass. Considering the popularity of oak forests both in Poland and in Europe and the fact that biomass residues may be a ubiquitous and easily accessible source of biochar, the authors were the first to attempt to characterize and compare chars from different parts of oak biomass collected at the same time and coming from one area. The aim of the research was to broadly analyze and compare the physicochemical and calorific properties of raw biomass from different parts of oak (wood, bark, brushwood, leaves and acorns) and to evaluate the impact of the pyrolysis process on these properties. The authors found it important and innovative to study the explosive index of individual chars in order to obtain detailed knowledge on the production, storage, transport and use of biocarbon materials. The novelty of this work is based on the comparison of the physicochemical properties of pyrolysates obtained from different types of oak biomass and the possibility of developing a method based on the presented and future research in order to obtain functional pyrolysates. The results presented in the paper below are the basis for further research in order to identify the best raw material derived from oak biomass for the production of functional pyrolysates, designed to meet the needs of soil and plants for specific nutrients.

## 2. Materials and Methods

### 2.1. Research Object

To produce biochar, wood, bark, branches, leaves and acorns of sessile oak (*Quercus petraea* (Matt.) Liebl.) were used separately. The material was collected in forests growing in the Carpathian Foothills, in southern Poland. The material intended for testing was brought to an air-dry state and then crushed.

### 2.2. Pyrolysis Process

The pyrolysis process was carried out using a retort furnace FCF 2R dedicated to thermal treatment in the atmosphere of inert gas, propertied a post-process gas cooler that had a water well (CZYLOK, Jastrzębie-Zdrój, Poland) [89].

**Figure 1:**
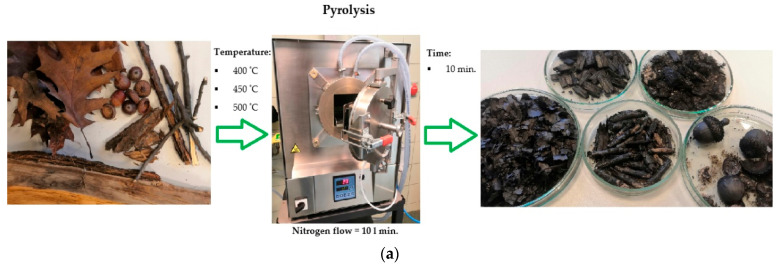

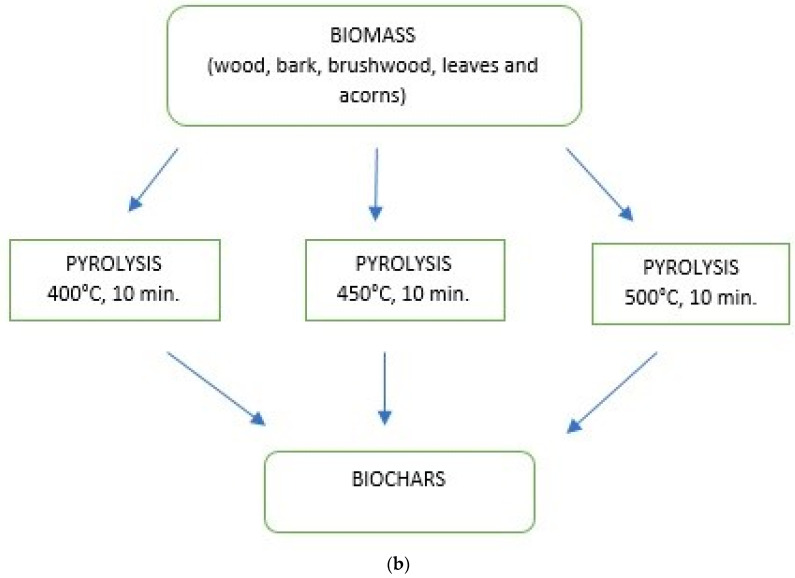
Scheme of the samples preparation process (**a**), flow chart of the experiment operation (**b**).

The pyrolysis of the tested materials was carried out at the following temperatures: 400, 450 and 500 °C. The residence time at the final temperature (nitrogen atmosphere with a purity of 99.99%, gas flow of 10 L/min) was 10 min (Figure 1). The pyrolysis temperature and the duration of the process were determined on the basis of numerous previous authors’ own research. For purification, the chars obtained in the process were sieved through a sieve with a hole diameter of 1 mm. Then the pyrolysates were rinsed with distilled water. The research material prepared in this way was dried for 12 h (temperature 80 °C) to remove potential contaminants.

### 2.3. Analysis of Samples

Basic physicochemical parameters for the tested samples were determined (proximate and ultimate analysis) along with the calorific value. The research used the thermogravimeter LECO TGA 701, an elementary composition analyzer of the truespec LECO CHN (Leco, St. Joseph, MI, USA) and LECO AC 500 isoperibolic calorimeter (Leco, St. Joseph, MI, USA). The dust explosiveness was measured with a KSEP20 device with a KSEP 310 control unit (Kuhner AG, Basel, Switzerland). The device consisted of a round test chamber with a volume of 20 dm^3^. The water jacket is responsible for the dissipation of explosive heat and the thermostatic control of the test temperatures [92].

The test dust was dispersed under pressure via an inlet valve. The inlet valve opened and closed pneumatically. The ignition point is located in the central part of the device—two chemical detonators with an energy of 5 kJ each. The device was equipped with pressure piezoelectric sensors from Kistler, which recorded the parameters of the process. Based on the obtained results, the highest explosion pressure P_max_ was determined. This is the highest noted outbreak pressure of the combustible mixture as a combustible material and air. Using the P_max_ parameter, and the read value of the highest pressure increase over time (*dp*/*dt*)_max_ *V*^1/3^, it was possible to determine the explosion class *K_st_*_max_. Explosion class *K_st_*_max_ was taken as a determinant of European standards, qualifying combustible dust according to EN 14034 [93]. The parameter was calculated using the following equation:

$$K\max=Kst=\sqrt[{3}]{V(\frac{dp}{dt}})\max=0.271(\frac{dp}{dt})\max[mbars^{-1}].$$

*K_st_*_max_ —explosivity index
*V*—volume of test chamber
(*dp*/*dt*)_max_—indicator of maximum explosion pressure gain.

The calculated value of the explosivity index are subjected to classification according to Table 1, where class St1—material with low explosiveness; class St2—material medium susceptible to the risk of explosion; class St3—material that is very susceptible to the risk of explosion.

The raw biomass and the obtained pyrolysates were subjected to laboratory tests compliance with current analytical standards (Table 2).

The yield of ash and volatile substances yield in the tested materials was made using a thermogravimetric method, with the use of the TGA 701 device by LECO (LECO Corporation, Saint Joseph, MI, USA). The TGA 701 analyzer is equipped with a 19-position autosampler and an automatic scale with a sensitivity of 0.0001 g. The device has a measurement accuracy of up to 0.02% and a temperature range of 20–1000 °C. The research procedure was based on placing 3-g weights in the measuring crucibles and selecting and setting the appropriate operating parameters of the device. Total ash was determined at the temperature of 600 °C—ashing under nitrogen atmosphere. Measurement of the total content of volatile substances with the use of thermogravimetric analysis consisted in evaporating water at the temperature of 105 °C, and then heating the tested material (nitrogen atmosphere) to the temperature of 950 °C using the crucible cover. The percentage of ash and volatile substances in the analyzed materials was calculated automatically using a computer application.

The percentages of total carbon, hydrogen and nitrogen were determined with a TrueSpec CHN analyzer by LECO (LECO Corporation, Saint Joseph, MI, USA). The LECO TrueSpec CHN analyzer is designed for the simultaneous determination of carbon, nitrogen and hydrogen. The basis of the device operation is based on the principles of the Dumas method, which is also called the method of high-temperature combustion in an oxygen atmosphere. This allows the content of the above-mentioned elements in the tested sample to be determined in no more than 5 min. The analysis process takes place in three stages—rinsing, incineration, and determination. Initially, the analyzed sample is “transported” to a sealed airlock, where the gases that appeared during the sample delivery are removed there (the gas system of the device is completely flushed). In the next stage, the test sample is transported to the ceramic crucible inside the combustion tube. The use of high temperature (950 °C) and pure oxygen flow enable very fast and effective combustion of the tested materials. The products of the combustion process go to the ballast tank, previously flowing through the filters, as well as a secondary furnace (full combustion of the material, removal of water vapor). In the last step, the carbon and hydrogen content is measured using infrared absorption detectors and nitrogen using a thermoconductive detector.

The calorific value was tested with an AC500 calorimeter by LECO (LECO Corporation, Saint Joseph, MI, USA). The principle of operation of the analyzer is to determine the heat of combustion of the sample in an oxygen atmosphere in a “calorimetric bomb” immersed in water. Controlling the heat transfer in all planes is possible thanks to the water jacket which completely surrounds the measuring system. The device is equipped with an electronic thermometer with a measuring accuracy of 0.0001 °C. Continuous temperature reading allows you to monitor the possible energy exchange in the system: tank surrounding the vessel—calorimetric vessel. A potential change in ambient temperature can thus be automatically corrected in the calculation of the final result. The computer software determines the difference in water temperature during the measurement, giving the result with the identification code and the weight used.

For each group of nine samples, the rotor of a digestion system was also filled with a blank sample. The samples were digested (0.1 g) at an algorithm of temperature increasing as specified for biological samples, never exceeding 220 °C. This procedure was carried out in an Ethos One microwave digestion system from Milestone. The vessels were opened after the mineralization process had been completed and the samples with acid had been brought to room temperature. Afterwards, they were replenished with water to a volume of 50 mL. The measurement of macro-, microelements and heavy metals content was performed on an ICP-OES spectrometer, a Thermo iCAP Dual 6500 with horizontal plasma, and with the capacity of detection being determined both along and across the plasma flame (radial and axial). Before measuring each batch of 10 samples, the equipment was calibrated with the use of certified Merck models, with concentrations of 10.000 ppm for Ca, Fe, K, Mg, and P and 1.000 ppm for Al, As, Cd, Cr, Cu, Mn, Mo, Na, Ni, Pb, S, Sr and Zn. The measurement result for each element was adjusted to account for the measurement of elements in the blank sample. In each case, a 3-point calibration curve was used for each element, with optical correction in applying the method of internal models, in the form of yttrium and ytterbium ions, at concentrations of 2 mg L^−1^ and 5 mg L^−1^, respectively. The analytical methods were validated using two independent tests. The detection threshold achieved for each tested element was equal to or higher than 0.01 mg kg^−1^.

### 2.4. Names of the Materials Tested

To facilitate the further identification of analyzed samples, the samples were marked with symbols according on the type of raw material, temperature and duration of the pyrolysis process:

A—oak wood
B—oak bark
C—oak branches
D—oak leaves
E—oak acorns
0—heat raw material
1—pyrolysis (temp. 400 °C; 10 min.);
2—pyrolysis (temp. 450 °C; 10 min.);
3—pyrolysis (temp. 500 °C; 10 min.);

For instance, A0—thermally unprocessed oak wood, B2—oak bark subjected to pyrolysis at 400 °C and a residence time of 10 min.

### 2.5. Statistical Analysis

The influence of the research factors imaged by the selected properties, and the relationships between these factors, were analyzed using Analysis of Variance (ANOVA) program using the Duncan test. Statistical calculations were performed using the Statistica 12 computer software. A significance threshold of ≤0.05 was accepted for all performed analyses. The obtained results were analyzed individually for each type of materials and the number of repetitions *n* = 3 [99,100].

## 3. Results and Discussion

The use of pyrolysis and the increase in its temperature caused an increase in the ash yield and carbon while decreasing the content of hydrogen and volatile substances [96]. Table 3 shows the differences in total carbon, total nitrogen, hydrogen, ash yield and volatile substances between raw samples. The following table also shows the effect of using different pyrolysis temperatures on pyrolysis parameters. Among the raw oak-derived biomass analyzed, the highest carbon content was characterized by oak leaf biomass: 51.56%, a slightly lower content of this element was determined in oak branches: 50.41%. Biomass from wood and oak bark showed almost identical carbon content—49.86 and 49.87%. The lowest concentration of total carbon among the analyzed biomass was characterized by acorns, with an average carbon content of 40.45%. In the analyzed pyrolysates, the highest carbon content was characterized by oak wood samples subjected to pyrolysis at 450 and 500 °C in 10 min—an increase of more than 66% compared to the raw material was recorded. The largest increase in total carbon concentration in pyrolysates relative to crude biomass was recorded in acorn samples. Pyrolysates from acorns formed at 450 and 500 °C in 10 min achieved more than 100% increase in total carbon concentration. The lowest increase in carbon concentration after the pyrolysis process was recorded for oak bark samples. Pyrolysis at 450 and 500 °C in 10 min resulted in an increase in carbon concentration by only 20%. The increase in total carbon content for leaf and branch pyrolysates at the two highest temperatures oscillated at about 40%. The higher pyrolysis temperature results in a greater increase in total carbon concentrations in the tested materials. The lowest increase in total carbon concentration occurred during pyrolysis at 400 °C and a duration of 10 min. Pyrolysis temperatures of 450 and 500 °C provided very similar effects in the form of an increase in total carbon concentration. The results obtained are consistent with reports from the literature, that as the charring temperature increases ash yield and total carbon in the material and at the same time there is a decrease in the content of hydrogen and volatile substances [101]. High carbon content suggests that biochars probably still contains a certain amount of original organic plant residues such as cellulose. Increased carbon content along with an increase in pyrolysis temperature occurs due to a higher degree of polymerization, leading to a more condensed carbon structure in the biochar [102]. For example, the carbon content of orange pomace biochar increased with increasing pyrolysis temperature (ranging from 56.8 to 68.1%) [103]. Whereas Cantrell et al. observed that the carbon content of poultry litter biochar decreased with increasing pyrolysis temperature (ranging from 27.0 to 35.5%) [104]. Enders et al. conducting pyrolysis of oak and pine wood at temperatures of 300, 400, 500 and 600 °C observed significant changes in the carbon content obtained in pyrolysates. Scientists recorded a maximum value of carbon in pyrolysates at 75% [105]. Enders reports that the total C content of maize, hazelnut, oak and pine biomass ranged within 43–49%, while the C content in the obtained pyrolysates varied within 60–91%. During the study, the researchers observed a greater variability in the content of the element due to the pyrolysis temperature rather than from the type of raw material [105]. Kazimierski and Kardas showed that higher pyrolysis temperature influences an increase in the carbon content in pellets [106]. Saletnik et al. analyzed the effect of pyrolysis parameters on the carbon and nitrogen content of produced biochars in their previous studies. The highest content of carbon and nitrogen was characterized by carbonizates formed in pyrolysis with parameters of 400 °C and a time of 10 min. Biochars from willow wood chips showed the highest levels of total carbon: 73.6%, of rye and rapeseed straw, these values were accordingly: 69.5 and 59% [32]. Kratophile et al. state that pyrolysates obtained from straw and wood chips at 350 °C have carbon content of 64 and 74%, respectively, and nitrogen 1.3 and 0.3% [107]. Ulusal et al., in studies on pyrolysis of oak sawdust, showed the carbon content of carbonizates for a time of 30 min and temperatures of 400, 500 and 600 °C respectively: 81.64; 89.90; 92.36% [38]. Among the materials analyzed, only biomass from oak bark showed nitrogen content. The maximum concentration of nitrogen in the raw cortex was 0.32%, and in the resulting pyrolysates the values were very close to 400 °C—0.54%; 450 °C—0.54% and 500 °C—0.53%. In the remaining biochars, the content of this element was not recorded. Ulusal et al., researching pyrolysates from oak sawdust, recorded low concentrations of nitrogen in pyrolysates processed for 30 min. at 400, 500 and 600 °C 0.67, 0.69 and 0.79% respectively [38]. Saletnik et al. recorded a maximum concentration of nitrogen in carbonizates obtained from rye straw—1.1%, and rapeseed straw and willow chips of 1.9% [108].

Numerous reports of an increase in carbon content and a simultaneous decrease in total hydrogen content occurring with increasing pyrolysis temperature can be found in the literature. The results of the analysis are consistent with literature reports. There has been a decrease in total hydrogen content with increasing pyrolysis temperature. At temperatures of 450 and 500 °C in acorn carbonizates there was a decrease in hydrogen by less than 20% in the remaining carbonizates the hydrogen content decreased by approx. 50%. The share of volatile parts determines the course of the fuel combustion process, including the ease of its ignition. Fuels with low volatile yield are more difficult to ignite [109]. In all the pyrolysates tested, there was a decrease in the percentage of volatile substances in relation to biomass. The highest content of volatile substances was recorded for oak bark pyrolysates: 450 °C—33.61% and 500 °C—33.72% and for acorn pyrolysates 33.54% and 33.50%, respectively. Oak bark pyrolysates showed the greatest decrease in volatile substances relative to biomass, which had a volatile yield of 75.43%. The results obtained by the authors comply with those available in the subject literature. Tong et al. specified the volatile substance content of unprocessed biomass (wood, straw and forest residues) at 75–85% [110]. This value is consistent with the VMCs determined by Dyjakon et al. in horse chestnut, acorns and spruce cones. An increase in temperature causes a, decrease in VMC. The average volatile yield of carbon is 40% [15,111,112]. Heat-treated forest biomass is becoming very close to carbon in terms of volatile substances [15]. The literature reports that the loss of mass through the release of volatile substances released during thermic decomposition in the pyrolysis process results in a significant increase ash yield of the final product. The higher the temperature of the process, the greater the loss of volatile substances. This results in a greater increase in the percentage of ash in the material [15]. In the conducted study, as the temperature of the pyrolysis process increases, the ash yield of the analyzed carbonizates increases. The ash in biochars varied in the range of 4.29 to 12.96%. The highest gain was recorded in pyrolysates obtained from oak branches and oak bark. Pyrolysate from oak branches were characterized by the highest ash yield, for temperatures of 400, 450 and 500 °C respectively: 11.19; 12.88; 12.96%. The lowest ash concentration was recorded for pyrolysates from acorns i.e., 3.0, 4.88 and 4.92 respectively for pyrolysis temperatures: 400, 450 and 500 °C. The observed significant ash increase may result from the analysis method used and the occurrence of additional charcoal incineration. The literature on the subject also noted an increase in ash recovery after the pyrolysis process [38,92,102,113,114] Ash increase during the pyrolysis process has been observed by scientists in earlier studies. Ulusal et al. noted an increase in ash in willow sawdust depending on the temperature of pyrolysis (400, 500, 600 °C) and its time (15, 30, 60, 120 min.). Scientists noted that both the time of pyrolysis and the increase in the temperature of the process causes an increase in ash concentration. Ulusal has seen more than 4-fold increase in ash for all combinations of the process [38]. Scientists report that the increase in the ash content result from progressive concentration of inorganic constituents and OM combustion residues. Mineral matter forming ash remains in biochar following carbonization [102]. Studies using oak wood as a raw material for the production of biochar were reported. Scientists indicate that ash yield was below 1% by weight [113]. Charvet et al. analyzed carbonizes from different wood species produced at 400 °C. The results indicate that ash yield in charcoal is 2 to 3 times higher than in wood, which is consistent with the fact that most of the ash in the raw material remains in charcoal [114].

In Table 4 presents the characteristics of selected biochars derived from plant and waste biomass obtained at different pyrolysis temperatures [56,92,115,116,117,118,119,120,121,122,123,124,125,126]. The presented data comes from the literature of the subject and is consistent with the results obtained by the authors of the publication. The amount of carbon obtained in pyrolysates depends on the type of biomass and the temperature of the pyrolysis process. In the biocarbons presented in the table, the ash yield ranges from 0.7% to 64.5%. The highest ash yield among the presented literature data was recorded for oak wood—64.5% [119]. Definitely higher ash yield is found in biocarbons from waste biomass: chicken manure—55.3% [115]; pig manure—46.5% [118]; swine manure—49.8% [116]. Carbon content in the presented biocarbons ranged from 27.2 to 88.9%. The highest carbon content was characteristic for the pine chip—88.9% [121]. The lowest carbon content is characteristic of biochars from chicken manure waste biomass—27.2% [115]; pig manure—44.1% [118]; The physical and chemical properties of biochar are strongly correlated with the starting material (biomass) and the pyrolysis temperature. Both of these factors influence the function of biochar as an additive to soil [102].

The authors aimed to analyze macroelements in the studied biochars. The aim of the study was to select the optimal temperature of the pyrolysis process to obtain pyrolysates with the greatest variety of macroelements. Table 5 shows the concentrations of elements in the raw mass and in pyrolysates produced using a varied process temperature. As a result of the conducted analyzes, it can be noticed that the most favorable temperature of the pyrolysis process in terms of the macronutrient content in chars is the temperature of 500 °C. The conducted research shows that the so far unexplored pyrolysates from oak leaves are rich in macroelements. The lowest value of the sum of macronutrients was recorded for oak wood pyrolysates. These values were arranged in descending order as follows: leaves—6400.11 mg 100 g^−1^; acorns—5730.96 mg 100 g^−1^; bark—3415.60 mg 100 g^−1^; brushwood—3175.07 mg 100 g^−1^, wood—365.20 mg 100 g^−1^. These values for the temperature of 450 °C were as follows: leaves—6043.34 mg 100 g^−1^; acorns—5300.93 mg 100 g^−1^; bark—3297.97 mg 100 g^−1^; brushwood—2963.37 mg 100 g^−1^, wood—362.52 mg 100 g^−1^. The greatest variety of high macronutrients was characterized by pyrolysate obtained from oak leaves. High levels of Ca, Fe, K, Mg, P, S, Na were recorded therein. Pyrolysates from acorns showed high content of Fe, K, P and S. Oak bark biochars were rich in Ca, Fe and S. The highest concentration of phosphorus and potassium, 2756.16 and 2437.00 mg 100 g^−1^ respectively showed pyrolysates from acorns, whereas the highest concentration of magnesium was recorded for oak-leaf pyrolysates: 422.30 mg 100 g^−1^. The highest Ca content at 2482.14 mg 100 g^−1^ was characterized by pyrolysates from oak branches. The main ingredients important for soil supply with nutrients include Ca, K and Mg. It was reported that the high supply of Ca is typical of oak [127]. Biochars are abundant in mineral elements such as Na, K, Ca, Fe and Mg. Their concentrations vary with the type of biomass and with the pyrolysis temperature. The highest levels of elements in biochar may vary depending on the temperature [102]—Ulusal et al. indicated nutrients in oak sawdust in the amount of: 0.74µg g^−1^ Na; 5.29 µg g^−1^ K; 108.8 µg g^−1^ Ca and 1.81 µg g^−1^ Mg [38]. The increases in Mg, Ca, K, and P on biochars pyrolyzed at high temperatures as being due to increased ash content (ranging from 4.0 to 33.1%). Biochars with high ash contents also tend to have greater amounts of PAHs and trace metals [102] Naeem et al. noted in their research that the general trend regarding elements: P, Si, S, K, Ca, Mg, Fe, Cu, Zn and Mn is that pyrolysis temperature increases, the content of these elements in biochar increases, but their bioavailability decreases. As they report, at a higher temperature, these elements are incorporated into the highly aromatic structure of biocarbon [128]. The pyrolysis temperature and processing time are reported to have a great influence on the chemical composition of biochar. The inorganic fraction in biochar i.e., metal compounds or minerals affect agronomic properties e.g., organic compounds may affect the mechanisms between biochar and soil [39,129]. Ulusal reports that the biochar obtained from oak sawdust contained nutrients that have a beneficial effect on soil fertility. Scientists report that the increase in temperature and process time increased each nutrient. Processing time was a more effective parameter than temperature in increasing Na, Mg and Ca [38]. According to Deng et al. K, Ca, Mg, Na, Si, Fe and Al are the dominant elements in sewage sludge biochars (SSB) from pyrolysis or co-pyrolysis [130]. Previous studies have shown that inorganic elements are often retained in SSB after pyrolysis because they do not decompose or volatilize at pyrolysis temperatures of 400–600 °C [58]. The P content of plants ranges from 0.1% to 1.0%. Pyrolysis converts organic P into inorganic P, resulting in biochar enrichment with phosphorus. Biochar enriched in P can be a source of P for plant growth. In addition, phosphorus in biochars can bind some heavy metals through precipitation [131]. Phosphorus in the early stages of plant development allows proper growth of the root system, while calcium is an important factor regulating cellular metabolism, performs a structural function and is a universal carrier of information [132,133,134]. Potassium, immediately after nitrogen, is the fastest absorbed element by plants, especially young ones with rapidly growing meristematic tissue from which they are made roots and stems [135]. This element is one of the most important nutrients for plants; in conditions of deficiency, it is directed first to growth cones and young leaves [132]. The use of biochar can improve the fertility of problematic soils. This is because biochar is considered an organic fertilizer containing organic C and plant nutrients such as N, P, K, Ca, Mg, S, Fe, Mn, Cu, Zn and Si. Depending on the nutrient deficiency in problem soils, biochar may be coated to meet plant needs for specific nutrients. If there are no suitable raw materials for specific nutrients, biochar can be designed so that it meets the demand. A characteristic feature of biochar fertilizers is the slow release of nutrients, mediated by unique biochar structures and sorption and desorption process [136].

The aim of the research was to analyze the presence of heavy metals in the preserved oak biomass pyrolysates. The results obtained are summarized in the Table 6. Among the quality requirements for biochar, the level of pollutants such as heavy metals is considered crucial for the safe introduction into soils [60]. The degree and purity of biochar methods of production and feedstock has the capacity to influence heavy metals. Biochar may contain heavy metals (HMs), which include copper, zinc, nickel, lead, chromium, manganese [12]. Because of the occurrence of several functional groups on the biochar surface for example COO and OH, biochar form complexes with heavy metals, which results in their immobilization and a decrease in bioavailability. The presence of heavy metals in biocarbon is depends on the feedstock used and the duration and temperature of pyrolysis. Pyrolysis conditions greatly affect nutrient properties contents and so biochar should be tested on a batch-by-batch basis to determine specific properties [1].

In the examined pyrolysates, the content of aluminum and molybdenum, as well as chromium, was not recorded (regarding chromium, the exception was acorn chars, in which an increase in this element was recorded along with an increase in the temperature of the process). As a result of the tests, an increase in the content of arsenic, cadmium, copper, nickel (the exception was a decrease in concentrations in oak wood) and lead (not detected in acorns). However, the concentration level of labeled heavy metals in pyrolysates was very low, not exceeding the acceptable standards set by the quality standards for biochar [60]. The subject literature reports that the pyrolysis process promotes changes in the chemical speciation and characteristics of the bio-carbon matrix, leading to a decrease in bioavailable fractions of heavy metals in biochar. For example, Hossain et al. noted the accumulation of heavy metals in biochar and a marked decrease in available heavy metal content [137]. Jin et al. found that rapid pyrolysis significantly inhibited the leaching of heavy metals from biochar [138]. In addition, Agrafioti et al. found that pyrolysis inhibits the release of heavy metals in acetic acid extraction at pH 5.9 and 6.0 [139]. Subject literature reports that heavy metals are stationary and stable in biochar and the pyrolysis process may inhibit their release to the soil [140].

**Figure 2:**
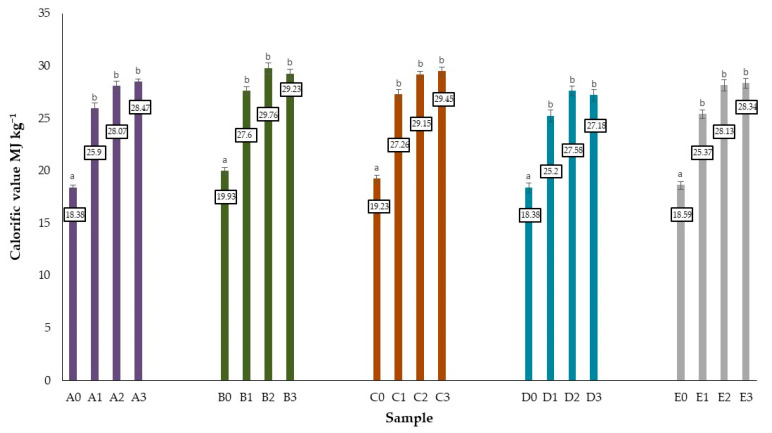
Calorific value of oak biomass and produced biochars. Differences between average values described with the same alphabet signs are statistically insignificant at the level of *p* ≤ 0.05 based on the Duncan test. The data were analyzed separately for each type of materials.

The authors aimed to compare the calorific value of the tested biomass and pyrolysates obtained at different temperatures of the pyrolysis process. Figure 2 shows the average calorific values of the tested samples. A significant increase was noted in calorific value for pyrolyzed samples. The highest calorific value among the raw biomass tested was characterized by oak bark i.e., 19.93 MJ kg^−1^, slightly lower values for oak branches 19.23 MJ kg^−1^, followed by acorns 18.57 MJ kg^−1^, the lowest calorific value was recorded for oak wood and oak leaves 18.38 MJ kg^−1^. The pyrolysis process influenced the increase in calorific value of the analyzed biomass. Pyrolysis at 400 °C resulted in an increase in the calorific value of biochar by approximately 40%. The process temperature of 450 and 500 °C resulted in an increase of more than 50%. The highest increase in the test parameter was recorded for pyrolyzates from acorns formed at a process temperature of 500 °C, this was an increase of 53% compared to the control sample. The highest calorific value among the tested pyrolyzates was recorded for bark pyrolyzates obtained at 500 °C, bark pyrolysates obtained at 450 °C had a slightly lower value, 29.76 and 29.23 MJ kg^−1^ respectively. Approximate values were obtained for pyrolyzates from oak branches: 29.15 and 29.45 MJ kg^−1^ respectively. The calorific value is the basic characteristic of the fuel and its properties. The higher the calorific value the greater the thermal energy yield during the combustion of the material [15]. The results obtained are similar to those described by the subject literature. Dyjakon et al. observed an increase in calorific value of chestnuts, oak acorns and spruce cones due to thermal conversion [15]. Saletnik et al. analyzed the calorific value from the raw biomass of fruit trees, i.e., apple, cherry, and pear branches, and from biochars produced using this type of biomass during pyrolysis processes conducted under various conditions. The plant biomass was thermally processed at 400, 450, or 500 °C for a duration of 5, 10, or 15 min. It was found that the mean calorific value of all of the biochars was increased by 62.24% compared to the non-processed biomass. More specifically, the mean calorific values of the biochars produced from apple, cherry, and pear branches amounted to 27.90, 28.75, and 26.84 MJ kg^−1^, respectively [92]. Charvet et al. analyzing wood derived from different species proved that charcoal exhibits significantly higher calorific values (LHV) compared to raw wood: from 16.4–19.0 MJ kg^−1^ for dry raw wood to 26.7–29.0 MJ kg^−1^ for dry charcoal, which represents an increment of approximately 50%. In this study, researchers recorded an increase in caloric content for oak wood from 17.1 to 26.7 MJ kg^−1^ for oak wood pyrolysates obtained at 400° C. The caloric content of the obtained biochar corresponds to approximately 80–90% of LHV graphite (32.8 MJ kg^−1^ [141]), which shows that high quality carbonization is formed without the need for complex conditions [114].

**Figure 3:**
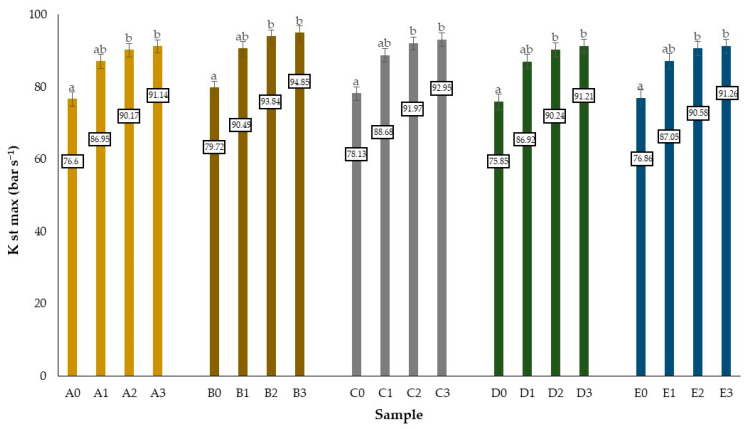
Explosivity indicator of dust of oak wood, bark, branches, leaves, acorns and produced biochars. Differences between average values described with the same alphabet signs are statistically insignificant at the level of *p* ≤ 0.05 based on the Duncan test. The data were analyzed separately for each type of materials.

In order to determine the safe use, production, storage and transport of the obtained biochars, the authors examined their explosive properties. The explosivity index *K_st_*_max_ calculated using a specific standard determines the immediate threat of dust explosion [142]. Analysis of the obtained data and the value of this parameter allows the classification of oak wood, bark, branches, leaves and acorns as well as biochar produced from this biomass into the first class of dust explosion danger (St1). This means that these materials are hardly susceptible to explosiveness. The explosivity index value obtained for wood, bark, branches, leaves and acorns respectively at 76.6; 79.72; 78.13; 76.6; 76.86 bar s^−1^. The explosivity index value for biochar samples was grew as the thermal temperature of the treatment higher. The maximum explosivity rate obtained among all analyzed samples was recorded in the case of oakbark biochar (500 °C, 10 min.) i.e., 94.85 bar s^−1^. In turn, the average value of this indicator for all biochar obtained regardless of pyrolysis parameters was 94.75 bar s^−1^ (Figure 3). Bajcar et al. showed an increase in the explosion index *K_st_*_max_, which, in the case of raw willow biomass, was estimated at the level of 72 bar s^−1^, and for the torrefied material amounted to 81 bar s^−1^. A similar tendency was identified in the case of wheat straw; the dust explosion index *K_st_*_max_ of raw biomass amounted to 55 bar s^−1^, and with the torrefied materials it increased to 62 bar s^−1^ [143]. Saletnik et al. classified the thermally unprocessed oak, coniferous pellets and their mixture, as well as the thermally processed forms obtained from them, into the first class of dust explosion hazard (St1)—a material not susceptible to explosiveness. Scientists noticed in-crease in this parameter for the obtained biocarbons with an increase of the temperature range and the duration of the pyrolysis process [144]. The present study shows that modifications of raw biomass required for the production of fuels with better quality parameters do not in-crease the risk of explosion. The observed tendency for an increase is associated with changes in the composition and physical structure of the material. The thermal processes leads to an increased concentration of carbon, higher contents of volatile substances, and greater brittleness observed in the materials after thermal treatment. Despite the visible trend, these differences are not significant and do not result in a change of dust classification [143]. According to Cashdollar, Cordero et al. and Demirbas, as well as other researchers, the differences between raw and thermally processed biomass can mainly be explained by the different emissivity of the respective materials linked to the mechanisms of heat transfer [145,146,147].

## 4. Conclusions

The article presents the thermal treatment of oak biomass (wood, bark, brushwood, leaves and acorns) for obtaining biochar as materials that can be used as fuel and fertilizer material. It has been found that the matrices of the obtained materials are rich in numerous macronutrients. Depending on the source of origin, biochar was characterized by different content of macronutrients. The conducted research shows that the so far unexplored pyrolysates from oak leaves are rich in macroelements. High levels of Ca, Fe, K, Mg, P, S, Na were recorded therein. Pyrolysates from acorns were high in Fe, K, P and S. Oak bark biochars were rich in Ca, Fe, S and contained nitrogen. The conducted research shows that the pyrolysis of oak biomass significantly increases the calorific value of the biomass, while maintaining the safety of its processing. The explosion classification of biocarbon dust in relation to the raw biomass has not changed. The average explosivity rate, *K_st_*_max_, for all biochar tested was 94.75 bar s^−1^.

This research provides a starting point for further analyses to design pyrolysates from oak biomass that would meet plant and soil needs for specific nutrients. It can be concluded that pyrolysis has the potential to add value to regionally available oak biomass on a sustainable basis and help to restore or improve essential soil functions. Biochar produced from oak biomass and applied as agricultural fertilizer can benefit the economy, especially in the areas of agriculture and forestry.

## 5. Future Perspective

The future prospect of using oak biochar to improve soil fertility and increase crop yields seems favorable. Biochar as a fertilizer for the soil lasts a long time and does not need to systematically be added to agricultural fertilizers, making it profitable. Biochar desorption properties depend on the pyrolyzed temperature, feedstock type, and the rate biochar application. It is believed that several biochar types should be able to accomplish different soil nutrients in the same soil or can be used differently in soils to obtain the anticipated nutrient supply effects. Therefore, there is a need for future research on the development of biochar as a fertilizer.

Future research should focus on optimizing production systems to produce optimized biochar products from oak biomass that can be used effectively to improve soil properties and to cater for plants with specific nutrients.

## Figures and Tables

**Table 1:** Dust explosion classes [94].

Explosion Class.	*K_st_*_max_ Value [bar s^−1^]
St1	≤200
St2	200–300
St3	>300

**Table 2:** Analyzed properties and research methods used.

Parameter	Research Method
Content of carbon, nitrogen and hydrogen	PN-EN 15104:2011 [95]
Ash	PN-EN 13775:2010 [96]
Content of volatile substances	PN-EN 15138:2011 [97]
Calorific value	PN-EN 13918:2010 [98]
Explosion index *K*_st max_	PN-EN 14034-2 [94]

**Table 3:** Contents of total nitrogen, total carbon, hydrogen, ash, and volatile substances in oak biomass and its biochars.

Sample	C	H	N	Ash	Volatile Substances
	%				
A0	49.87 ^a^ ± 0.37	6.19 ^b^ ± 0.02	<0.04	2.11 ^a^ ± 0.04	79.24 ^c^ ± 0.11
A1	78.91 ^b^ ± 0.90	3.49 ^a^ ± 0.05		9.41 ^b^ ± 0.07	38.92 ^b^ ± 0.09
A2	82.36 ^b^ ± 0.62	3.05 ^a^ ± 0.04		11.38 ^c^ ± 0.52	31.68 ^a^ ± 0.12
A3	83.13 ^b^ ± 0.95 ± 0.09	3.02 ^a^ ± 0.02		11.28 ^c^ ± 0.43	31.52 ^a^ ± 0.27
B0	49.86 ^a^ ± 0.07	6.49 ^b^ ± 0.03	0.32 ^a^ ± 0.03	2.24 ^a^ ± 0.06	75.43 ^c^ ± 0.08
B1	57.47 ^b^ ± 0.05	3.48 ^a^ ± 0.06	0.54 ^b^ ± 0.03	11.19 ^b^ ± 0.04	39.35 ^b^ ± 0.08
B2	60.96 ^b^ ± 0.20	3.05 ^a^ ± 0.05	0.54 ^b^ ± 0.03	12.88 ^c^ ± 0.03	33.61 ^a^ ± 0.11
B3	60.99 ^b^ ± 0.76	3.07 ^a^ ± 0.10	0.53 ^b^ ± 0.01	12.96 ^c^ ± 0.06	33.72 ^a^ ± 0.09
C0	50.41 ^a^ ± 0.50	6.74 ^b^ ± 0.03	<0.04	0.59 ^a^ ± 0.03	81.33 ^c^ ± 0.13
C1	70.03 ^b^ ± 0.32	3.78 ^a^ ± 0.10		3.37 ^b^ ± 0.06	38.56 ^b^ ± 0.08
C2	73.62 ^b^ ± 0.15	3.35 ^a^ ± 0.03		5.06 ^c^ ± 0.07	32.33 ^a^ ± 0.13
C3	73.52 ^b^ ± 0.21	3.32 ^a^ ± 0.08		5.05 ^c^ ± 0.03	32.50 ^a^ ± 0.07
D0	51.56 ^a^ ± 0.26	6.51 ^b^ ± 0.03		3.13 ^a^ ± 0.08	76.49 ^c^ ± 0.09
D1	68.76 ^b^ ± 0.16	3.73 ^a^ ± 0.10		9.51 ^b^ ± 0.13	36.91 ^b^ ± 0.18
D2	72.34 ^b^ ± 0.15	3.44 ^a^ ± 0.04		11.05 ^c^ ± 0.10	32.11 ^a^ ± 0.07
D3	72.41 ^b^ ± 0.08	3.44 ^a^ ± 0.10		11.08 ^c^ ± 0.05	32.21 ^a^ ± 0.04
E0	40.45 ^a^ ± 0.48	7.24 ^b^ ± 0.07		1.93 ^a^ ± 0.04	80.83 ^c^ ± 0.09
E1	77.82 ^b^ ± 0.41	5.87 ^a^ ± 0.03		3.00 ^b^ ± 0.06	38.74 ^b^ ± 0.12
E2	81.34 ^b^ ± 0.49	5.42 ^a^ ± 0.03		4.88 ^c^ ± 0.09	33.54 ^a^ ± 0.11
E3	81.40 ^b^ ± 0.35	5.34 ^a^ ± 0.08		4.92 ^c^ ± 0.09	33.50 ^a^ ± 0.07

Statistically significant differences marked by different letters (*p* ≤ 0.05). Differences between average values described with the same alphabet signs are statistically insignificant at the level of *p* ≤ 0.05 based on the Duncan test. The data were analyzed separately for each type of materials.

**Table 4:** Biochar characteristics in different temperatures from biomass.

Biochar	Pyrolysis Temperature	C	VM	Ash	References
	°C	%			
apple branches	400	70.22	28.46	5.04	Saletnik et al. [92]
apple branches	450	70.88	24.97	7.55	Saletnik et al. [92]
apple branches	500	73.54	21.71	7.09	Saletnik et al. [92]
cherry branches	400	75.19	31.85	4.69	Saletnik et al. [92]
cherry branches	450	77.26	27.75	4.89	Saletnik et al. [92]
cherry branches	500	80.66	22.72	5.45	Saletnik et al. [92]
pear branches	400	67.82	28.31	7.41	Saletnik et al. [92]
pear branches	450	69.19	24.51	8.76	Saletnik et al. [92]
pear branches	500	72.22	20.36	8.81	Saletnik et al. [92]
Coffee husk	450	61.3	26.2	12.9	Domingues et al. [115]
Chicken Manure	450	27.2	30.6	55.3	Domingues et al. [115]
Eucalyptus sawdust	450	78.6	28.5	0.7	Domingues et al. [115]
Sugarcane bagasse	450	81.6	24.0	2.1	Domingues et al. [115]
Pine bark	350	75.2	29.3	7.9	Lu et al. [56]
Swine manure	400	74.9	35.5	49.8	Jin et al. [116]
Rapeseed plant	400	71.3	27.1	12.2	Karaosmanoglu et al. [117]
Cow manure	400	60.2	27.4	15.3	Kolodynska et al. [118]
Pig manure	400	44.1	19.1	46.5	Kolodynska et al. [118]
Oak wood	450	71.3	15.6	64.5	Mohan et al. [119]
Corn cobs	500	77.6	-	13.3	Mullen et al. [120]
Corn stover	500	57.3	-	32.8	Mullen et al. [120]
Poultry litter	500	48.3	17.7	41.9	Novak et al. [121]
Pine chip	500	88.9	22.4	2.6	Novak et al. [121]
Corn stover	400	64.0	45.5	12.5	Rafiq et al. [122]
Corn stover	500	64.5	338	18.7	Rafiq et al. [122]
Black wattle	475	66.5	-	4.8	Uras et al. [123]
Sugarcane bagasse	475	57.3	-	12.1	Uras et al. [123]
Vineyard prunings	475	66.5	-	8.1	Uras et al. [123]
Tree barks	400	80.0	-	-	Venegas et al. [124]
Bamboo	450	76.9	-	-	Yao et al. [125]
Buckwheat husk	450	76.5	-	25.4	Zama et al. [126]
Mulberry wood	450	70.8	-	7.7	Zama et al. [126]
Peanut shells	450	70.8	-	16.9	Zama et al. [126]

**Table 5:** Content of selected macronutrients in oak biomass and its biochars.

Sample	Macronutrients						
	Ca	Fe	K	Mg	Na	P	S
	mg 100 g^−1^						
A0	113.35 ^a^ ± 2.41	8.34 ^a^ ± 1.31	91.25 ^a^ ± 3.63	11.30 ^c^ ± 0.17	12.85 ^c^ ± 1.69	163.8 ^c^ ± 3.58	24.14 ^c^ ± 0.11
A1	115.32 ^a^ ± 3.21	7.24 ^a^ ± 2.00	116.39 ^b^ ± 2.14	9.21 ^b^ ± 0.19	6.21 ^b^ ± 0.48	136.03 ^b^ ± 1.12	14.45 ^b^ ± 0.63
A2	115.83 ^a^ ± 3.56	6.19 ^a^ ± 2.00	128.43 ^c^ ± 3.08	8.87 ^b^ ± 0.17	3.26 ^a^ ± 0.34	106.13 ^a^ ± 0.67	4.44 ^a^ ± 0.35
A3	124.26 ^a^ ± 2.42	5.89 ^a^ ± 1.24	132.12 ^c^ ± 1.18	7.67 ^a^ ± 0.14	2.92 ^a^ ± 0.12	98.23 ^a^ ± 1.47	4.01 ^a^ ± 0.28
B0	880.33 ^a^ ± 17.61	4.17 ^a^ ± 2.03	190.81 ^a^ ± 4.53	49.45 ^a^ ± 1.36	11.05 ^c^ ± 0.26	423.92 ^a^ ± 3.91	92.68 ^c^ ± 0.74
B1	2269.85 ^b^ ± 71.63	15.19 ^b^ ± 2.06	232.31 ^b^ ± 5.22	84.39 ^b^ ± 2.21	8.26 ^b^ ± 0.10	456.12 ^a b^ ± 9.28	63.19 ^b^ ± 0.48
B2	2413.92 ^b^ ± 70.59	26.29 ^c^ ± 2.25	258.42 ^c^ ± 7.26	139.72 ^c^ ± 2.64	5.16 ^a^ ± 0.23	480.75 ^a b^ ± 12.78	54.31 ^a^ ± 0.42
B3	2482.14 ^b^ ± 69.28	32.13 ^c^ ± 1.65	252.13 ^c^ ± 7.07	164.52 ^d^ ± 3.01	4.42 ^a^ ± 0.31	512.39 ^b^ ± 8.95	49.57 ^a^ ± 0.38
C0	265.167 ^a^ ± 9.45	<0.01	109.34 ^b^ ± 1.09	39.55 ^a^ ± 0.70	4.82 ^a^ ± 1.33	510.5 ^a^ ± 2.11	25.61 ^a^ ± 0.30
C1	1468.20 ^b^ ± 85.27	6.21 ^a^ ± 0.75	87.15 ^a^ ± 4.62	68.08 ^b^ ± 3.19	5.28 ^a^ ± 0.39	657.72 ^b^ ± 1.91	27.81 ^a^ ± 0.19
C2	1878.92 ^b^ ± 106.12	8.00 ^a^ ± 1.90	107.05 ^b^ ± 5.53	148.00 ^c^ ± 3.43	9.48 ^b^ ± 0.60	819.92 ^c^ ± 0.95	32.61 ^b^ ± 0.29
C3	1952.36 ^b^ ± 101.47	9.14 ^a^ ± 1.23	111.09 ^b^ ± 5.83	192.46 ^d^ ± 2.93	12.41 ^c^ ± 0.62	906.75 ^d^ ± 1.83	34.12 ^b^ ± 0.26
D0	915.167 ^a^ ± 36.13	3.30 ^a^ ± 0.47	179.23 ^a^ ± 4.34	120.03 ^a^ ± 2.56	9.08 ^a^ ± 0.23	814.5 ^a^ ± 8.94	92.33 ^a^ ± 0.48
D1	1741.26 ^b^ ± 39.82	17.54 ^b^ ± 0.52	1038.62 ^b^ ± 33.27	262.61 ^b^ ± 8.38	10.63 ^b^ ± 0.28	1465.17 ^b^ ± 16.31	100.61 ^a b^ ± 0.38
D2	2058.42 ^b^ ± 121.94	29.97 ^c^ ± 0.76	1152.58 ^b^ ± 43.46	382.17 ^c^ ± 12.11	11.00 ^b^ ± 0.30	2439.17 ^c^ ± 7.42	104.01 ^b^ ± 0.00
D3	2132.43 ^b^ ± 78.42	31.49 ^c^ ± 2.18	1174.43 ^b^ ± 46.15	422.30 ^c^ ± 10.16	11.61 ^b^ ± 0.54	2659.34 ^c^ ± 21.29	111.78 ^b^ ± 5.62
E0	119.88 ^a^ ± 1.96	<0.01	576 ^a^ ± 14.93	42.38 ^a^ ± 1.27	3.33 ^b^ ± 0.42	612.5 ^a^ ± 4.13	38.81 ^a^ ± 0.37
E1	279.32 ^b^ ± 7.28	19.61 ^a^ ± 1.15	1753 ^b^ ± 41.92	67.95 ^b^ ± 0.94	2.68 ^b^ ± 0.08	2019.37 ^b^ ± 9.97	49.76 ^b^ ± 0.21
E2	347.92 ^b^ ± 4.26	29.43 ^b^ ± 0.71	2397 ^c^ ± 37.79	127.48 ^c^ ± 1.31	2.28 ^a^ ± 0.17	2426.25 ^c^ ± 7.15	62.86 ^c^ ± 0.28
E3	381.16 ^b^ ± 8.22	33.79 ^b^ ± 2.58	2437 ^c^ ± 46.51	154.67 ^d^ ± 3.47	1.97 ^a^ ± 0.14	2756.16 ^d^ ± 8.91	68.94 ^c^ ± 0.19

Statistically significant differences marked by different letters (*p* ≤ 0.05). Differences between average values described with the same alphabet signs are statistically insignificant at the level of *p* ≤ 0.05 based on the Duncan test. The data were analyzed separately for each type of materials.

**Table 6:** Content of selected micronutrients and heavy metals in oak biomass and its biochars.

Sample	Microelements										
	Al	As	Cd	Cr	Cu	Mo	Ni	Pb	Mn	Sr	Zn
	mg 100 g^−1^										
A0	<0.01	<0.01	<0.01	1.04 ± 0.06	<0.01	<0.01	0.57 ^d^ ± 0.03	<0.01	9.05 ^a^ ± 0.09	2.94 ^c^ ± 0.00	0.10 ± 0.00
A1		0.07 ^a^ ± 0.03		<0.01	0.04 ^a^ ± 0.00		0.35 ^c^ ± 0.02	0.02 ^a^ ± 0.01	13.11 ^b^ ± 0.07	0.40 ^b^ ± 0.02	<0.01
A2		0.11 ^a^ ± 0.07	0.02 ^a^ ± 0.01		0.10 ^b^ ± 0.03		0.15 ^b^ ± 0.00	0.04 ^a^ ± 0.03	17.06 ^c^ ± 0.15	0.40 ^b^ ± 0.02	
A3		0.14 ^a^ ± 0.04	0.04 ^a^ ± 0.01		0.12 ^b^ ± 0.01		0.10 ^a^ ± 0.02	0.05 ^a^ ± 0.03	21.42 ^d^ ± 0.19	0.01 ^a^ ± 0.00	
B0		<0.01	0.07 ^a^ ± 0.01		0.13 ^a^ ± 0.02		0.07 ^a^ ± 0.00	0.56 ^a^ ± 0.03	71.9 ^a^ ± 0.52	1.71 ^a^ ± 0.04	0.63 ^a^ ± 0.00
B1		0.11 ^a^ ± 0.03	0.10 ^a^ ± 0.02		0.45 ^b^ ± 0.03		0.19 ^b^ ± 0.02	0.69 ^b^ ± 0.02	119.49 ^b^ ± 1.60	3.19 ^b^ ± 0.10	1.01 ^b^ ± 0.09
B2		0.14 ^a b^ ± 0.06	0.11 ^a^ ± 0.03		0.85 ^c^ ± 0.03		0.29 ^c^ ± 0.00	0.82 ^c^ ± 0.02	173.98 ^c^ ± 1.64	4.89 ^c^ ± 0.12	1.38 ^c^ ± 0.00
B3		0.21 ^b^ ± 0.03	0.12 ^a^ ± 0.03		1.01 ^d^ ± 0.07		0.33 ^d^ ± 0.05	0.89 ^c^ ± 0.06	191.28 ^c^ ± 1.04	5.12 ^c^ ± 0.16	1.64 ^d^ ± 0.03
C0		0.05 ± 0.08	0.04 ^a^ ± 0.00		<0.01		0.04 ^a^ ± 0.01	0.02 ^a^ ± 0.03	107.6 ^a^ ± 0.70	0.57 ^a^ ± 0.01	0.64 ^a^ ± 0.00
C1		<0.01	0.08 ^b^ ± 0.01		0.58 ^a^ ± 0.02		0.17 ^b^ ± 0.02	0.12 ^b^ ± 0.00	329.83 ^b^ ± 1.68	2.37 ^b^ ± 0.26	1.98 ^b^ ± 0.01
C2			0.14 ^c^ ± 0.00		0.78 ^b^ ± 0.01		0.35 ^c^ ± 0.02	0.23 ^c^ ± 0.03	508.33 ^c^ ± 0.80	4.17 ^c^ ± 0.15	3.31 ^c^ ± 0.01
C3			0.18 ^c^ ± 0.04		0.91 ^c^ ± 0.03		0.41 ^c^ ± 0.07	0.28 ^c^ ± 0.03	564.96 ^c^ ± 2.12	4.93 ^c^ ± 0.18	3.82 ^c^ ± 0.07
D0			0.03 ^a^ ± 0.00		0.32 ^a^ ± 0.02		0.16 ^a^ ± 0.02	0.05 ^a^ ± 0.01	639.42 ^a^ ± 1.01	1.01 ^a^ ± 0.02	1.26 ^a^ ± 0.00
D1			0.06 ^b^ ± 0.03	0.01 ^a^ ± 0.00	1.68 ^c^ ± 0.01		0.22 ^a^ ± 0.04	0.08 ^a^ ± 0.02	982.55 ^b^ ± 1.23	2.08 ^b^ ± 0.03	3.09 ^b^ ± 0.02
D2			0.11 ^c^ ± 0.02	0.01 ^a^ ± 0.00	1.34 ^b^ ± 0.00		0.28 ^b^ ± 0.00	0.15 ^b^ ± 0.03	1421.25 ^c^ ± 3.28	2.68 ^c^ ± 0.08	4.86 ^c^ ± 0.02
D3			0.13 ^c^ ± 0.03	0.01 ^a^ ± 0.00	1.44 ^b^ ± 0.04		0.34 ^c^ ± 0.03	0.19 ^b^ ± 0.03	1728.39 ^d^ ± 2.41	3.01 ^c^ ± 0.23	5.12 ^c^ ± 0.32
E0		0.09 ^a^ ± 0.05	<0.01	<0.01	0.33 ^a^ ± 0.03		0.11 ^a^ ± 0.00	<0.01	77.52 ^a^ ± 0.50	0.08 ^a^ ± 0.00	0.18 ^c^ ± 0.00
E1		0.02 ^a^ ± 0.03	0.01 ^a^ ± 0.01	1.04 ^a^ ± 0.13	0.42 ^b^ ± 0.01		0.89 ^b^ ± 0.03		94.32 ^b^ ± 0.72	0.33 ^b^ ± 0.02	0.12 ^b^ ± 0.01
E2		0.05 ^a^ ± 0.03	0.02 ^a^ ± 0.00	1.26 ^a^ ± 0.19	0.54 ^c^ ± 0.02		1.34 ^c^ ± 0.00		104.42 ^b^ ± 1.40	0.43 c ± 0.00	0.12 ^b^ ± 0.01
E3		0.05 ^a^ ± 0.01	0.02 ^a^ ± 0.02	1.48 ^b^ ± 0.15	0.58 ^c^ ± 0.02		1.84 ^d^ ± 0.07		118.61 ^c^ ± 1.15	0.49 ^d^ ± 0.01	0.07 ^a^ ± 0.01

Statistically significant differences marked by different letters (*p* ≤ 0.05). Differences between average values described with the same alphabet signs are statistically insignificant at the level of *p* ≤ 0.05 based on the Duncan test. The data were analyzed separately for each type of materials.

## Data Availability

Not applicable.

## Abbreviations

LHV	Lower heating value
OM	Organic matter
SSB	Sewage sludge biochars
VMC	Content of volatile substances

## References

[1] Oni B.A., Oziegbe O., Olawole O.O. (2019). Significance of biochar application to the environment and economy. Ann. Agric. Sci..

[2] IBI Biochar Standards—Standardized Product Definition and Product Testing Guidelines for Biochar That Is Used in Soil, v.2.1. http://www.biochar-international.org/sites/default/files/IBI_Biochar_Standards_V2.1_Final.pdf.

[3] Chen B., Yuan B., Qian L. (2012). Enhanced bioremediation of PAH-contaminated soil by immobilized bacteria with plant residue and biochar as carriers. J. Soils Sediments.

[4] Singh N., Abiven S., Torn M.S., Schmidt M.W.I. (2012). Fire-derived organic carbon in soil turns over on a centennial scale. Biogeosciences.

[5] Quilliam R.S., Glanville H.C., Wade S.C., Jones D.L. (2013). Life in the ‘charosphere’—Does biochar in agricultural soil provide a significant habitat for microorganisms?. Soil Biol. Biochem..

[6] Perea-Moreno M.A., Samerón-Manzano E., Perea-Moreno A.J. (2019). Biomass as Renewable Energy: Worldwide Research Trends. Sustainability.

[7] Kilkis S., Krajacic G., Duic N., Rosen M.A., Al-Nimr M.A. (2018). Advancements in sustainable development of energy, water and environment systems. Energy Convers. Manag..

[8] Piekarczyk M., Kotwica K., Jaskulski D. (2011). Effect of spring barley straw ash on the chemical properties of light soil. Fragm. Agron..

[9] Li G., Hu R., Wang N., Yang T., Xu F., Li J., Wu J., Huang Z., Pan M., Lyu T. (2022). Cultivation of microalgae in adjusted wastewater to enhance biofuel production and reduce environmental impact: Pyrolysis performances and life cycle assessment. J. Clean. Prod..

[10] Huang Z., Zhang J., Pan M., Hao Y., Hu R., Xiao W., Li G., Lyu T. (2022). Valorisation of microalgae residues after lipid extraction: Pyrolysis characteristics for biofuel production. Biochem. Eng. J..

[11] Brewer C.E., Chuang V.J., Masiello C.A., Gonnermann H., Gao X., Dugan B. (2014). New approaches to measuring biochar density and porosity. Biomass Bioenergy.

[12] Al-Wabel M.I., Al-Omran A., El-Naggar A.H., Nadeem M., Usman A.R.A. (2013). Pyrolysis temperature induced changes in characteristics and chemical composition of biochar produced from cono-carpus wastes. Bioresour. Technol..

[13] Antar M., Lyu D., Nazari M., Shah A., Zhou X., Smith D.L. (2021). Biomass for a sustainable bioeconomy: An overview of world biomass production and utilization. Renew. Sustain. Energy Rev..

[14] Baldwin R.F. (2020). Forest Products Utilization within a Circular Bioeconomy. For. Prod. J..

[15] Dyjakon A., Noszczyk T. (2020). Alternative Fuels from Forestry Biomass Residue: Torrefaction Process of Horse Chestnuts, Oak Acorns, and Spruce Cones. Energies.

[16] Zan G., Wu T., Zhang Z., Li J., Zhou J., Zhu F., Chen H., Wen M., Yang X., Peng X. (2022). Bioinspired Nanocomposites with Self-Adaptive Stress Dispersion for Super-Foldable Electrodes. Adv. Sci..

[17] Zan G., Wu T., Zhu F., He P., Cheng Y., Chai S., Wang Y., Huang X., Zhang W., Wan Y. (2021). A biomimetic conductive super-foldable material. Matter.

[18] Sun Y., Sills R.B., Hu X., Seh Z.W., Xiao X., Xu H., Luo W., Jin H., Xin Y., Li T. (2015). A bamboo-inspired nanostructure design for flexible, foldable, and twistable energy storage devices. Nano Lett..

[19] Saletnik B., Zaguła G., Bajcar M., Tarapatskyy M., Bobula G., Puchalski C. (2019). Biochar as a Multifunctional Component of the Environment—A Review. Appl. Sci..

[20] Chhiti Y., Kemiha M. (2013). Thermal Conversion of Biomass, Pyrolysis and Gasification: A Review. Int. J. Eng Sci..

[21] Varma A.K., Shankar R., Mondal P., Sarangi P.K., Sonil N., Pravakar M. (2018). A Review on Pyrolysis of Biomass and the Impacts of Operating Conditions on Product Yield, Quality, and Upgradation. Recent Advancements in Biofuels and Bioenergy Utilization.

[22] Rhodes A.H., Carlin N.A., Semple O.R. (2008). Impact of black carbon in the extraction and mineralization of phenanthrene in soil. Environ. Sci. Technol..

[23] Li G., Bai X., Huo S., Huang Z. (2020). Fast pyrolysis of LERDADEs for renewable biofuels. IET Renew. Power Gener..

[24] Li G., Ji F., Bai X., Zhou Y., Dong R., Huang Z. (2019). Comparative study on thermal cracking characteristics and bio-oil production from different microalgae using Py-GC/MS. Int. J. Agric. Biol. Eng..

[25] Yang S., Wang S., Wang H. (2021). Particle-scale evaluation of the pyrolysis process of biomass material in a reactive gas-solid spouted reactor. Chem. Eng. J..

[26] Lu L., Pecha M.B., Wiggins G.M., Xu Y., Gao X., Hughes B., Shahnam M., Rogers W.A., Carpenter D., Parks J.E. (2022). Multiscale CFD simulation of biomass fast pyrolysis with a machine learning derived intra-particle model and detailed pyrolysis kinetics. Chem. Eng. J..

[27] Oyedeji O.A., Pecha M.B., Finney C.E., Peterson C.A., Smith R.G., Mills Z.G., Gao X., Shahnam M., Rogers W.A., Ciesielski N. (2022). CFD–DEM modeling of autothermal pyrolysis of corn stover with a coupled particle-and reactor-scale framework. Chem. Eng. J..

[28] Lewandowski W.M., Radziemska E., Ryms M., Ostrowski P. (2010). Modern methods of thermochemical biomass conversion into gas, liquid and solid fuels. Proc. ECOpole.

[29] Malińska K. (2012). Biochar-a response to current environmental issues. Eng. Prot. Environ..

[30] Saletnik B., Zaguła G., Saletnik A., Bajcar M., Puchalski C. (2020). Biochar and Ash Fertilization Alter the Chemical Properties of Basket Willow (*Salix viminalis* L.) and Giant Miscanthus (*Miscanthus x giganteu*s). Agronomy.

[31] Zadeh Z.E., Abdulkhani A., Aboelazayem O., Saha B. (2020). Recent insights into lignocellulosic biomass pyrolysis: A critical review on pretreatment, characterization, and products upgrading. Processes.

[32] Zhao L., Zhao Y., Nan H., Yang F., Qiu H., Xu X., Cao X. (2020). Suppressed formation of polycyclic aromatic hydrocarbons (PAHs) during pyrolytic production of Fe-enriched composite biochar. J. Hazard. Mater..

[33] Zhou H., Wu C., Onwudili J.A., Meng A., Zhang Y., Williams P.T. (2014). Polycyclic aromatic hydrocarbon formation from the pyrolysis/gasification of lignin at different reaction conditions. Energy Fuels.

[34] Lewandowski W.M., Ryms M., Kosakowski W. (2020). Thermal biomass conversion: A review. Processes.

[35] Zimmerman A.R., Gao B., Ahanna M.Y., Araujo J.R. (2011). Positive and negative carbon mineralization priming effects among a variety of biochar-amended soils. Soil Biol. Biochem..

[36] Downie A., Crosky A., Munroe P., Lehmann J., Joseph S. (2009). Physical properties of Biochar. Biochar for Environmental Management: Science and Technology.

[37] Suthar R.G., Wang C., Nunes M.C.N., Chen J., Sargent S.A., Bucklin R.A., Gao B. (2018). Bamboo Biochar Pyrolyzed at Low Temperature Improves Tomato Plant Growth and Fruit Quality. Agriculture.

[38] Ulusal A., Apaydın Varol E., Bruckman V.J., Uzun B.B. (2021). Opportunity for sustainable biomass valorization to produce biochar for improving soil characteristics. Biomass Convers. Biorefin..

[39] Han L., Ro K.S., Wang Y., Sun K., Sun H., Libra J.A., Xing B. (2018). Oxidation resistance of biochars as a function of feedstock and pyrolysis condition. Sci. Total Environ..

[40] Zhang Y., Ma Z., Zhang Q., Wang J., Ma Q., Yang Y., Luo X., Zhang W. (2017). Comparison of the physicochemical characteristics of bio-char pyrolyzed from moso bamboo and rice husk with different pyrolysis temperatures. BioResources.

[41] Conti R., Fabbri D., Vassura I., Ferroni L. (2016). Comparison of chemical and physical indices of thermal stability of biochars from different biomass by analytical pyrolysis and thermogravimetry. J. Anal. Appl. Pyrolysis.

[42] Windeatt J.H., Ross A.B., Williams P.T., Forster P.M., Nahil M.A., Singh S. (2014). Characteristics of biochars from crop residues: Potential for carbon sequestration and soil amendment. J. Environ. Manag..

[43] Wang J., Xiong Z., Kuzyakov Y. (2016). Biochar stability in soil: Meta-analysis of decomposition and priming effects. GCB Bioenerg..

[44] Crombie K., Mašek O., Sohi S.P., Brownsort P., Cross A. (2013). The effect of pyrolysis conditions on biochar stability as determined by three methods. GCB Bioenerg..

[45] Titiladunayo I.F., McDonald A.G., Fapetu O.P. (2012). Effect of temperaturę on biochar product yield from selected lignocellulosic biomass in a pyrolysis process. Waste Biomass Valor..

[46] Ghysels S., Rathnayake D., Maziarka P., Mašek O., Sohi S., Ronsse F. (2022). Biochar stability scores from analytical pyrolysis (Py-GC-MS). J. Anal. Appl. Pyrolysis.

[47] Zhang J., Liu J., Liu R. (2015). Effects of pyrolysis temperature and heating time on biochar obtained from the pyrolysis of straw and lignosulfonate. Bioresour. Technol..

[48] Chen D., Li Y., Cen K., Luo M., Li H., Lu B. (2016). Pyrolysis polygeneration of poplar wood: Effect of heating rate and pyrolysis temperature. Bioresour. Technol..

[49] Gurwick N.P., Moore L.A., Kelly C., Elias P. (2013). A systematic review of biochar research, with a focus on its stability in situ and its promise as a climate mitigation strategy. PLoS ONE.

[50] Suárez-Abelenda M., Kaal J., McBeath A.V. (2017). Translating analytical pyrolysis fingerprints to thermal stability indices (TSI) to improve biochar characterization by pyrolysis-GC-MS. Biomass Bioener..

[51] Fang Y., Singh B., Singh B.P. (2015). Effect of temperature on biochar priming effects and its stability in soils. Soil Biol. Biochem..

[52] Lee J., Sarmah A.K., Kwon E.E., Ok Y.S., Tsang D.C.W., Bolan N., Novak J.M. (2019). Production and formation of biochar. Biochar from Biomass and Waste Fundamentals Applications.

[53] Ronsse F., Bruckman V.J., Apaydın V.E., Uzun B.B., Liu J. (2016). Biochar production. Biochar: A Regional Supply Chain Approach in View of Climate Change Mitigation.

[54] Kambo H.S., Dutta A. (2015). A comparative review of biochar and hydrochar in terms of production, physico-chemical properties and applications. Renew Sustain. Energy Rev..

[55] Jien S.H., Ok Y.S., Tsang D.C.W., Bolan N., Novak J.M. (2019). Physical characteristics of biochars and their effects on soil physical properties. Biochar from Biomass and Waste Fundamentals Applications.

[56] Lu W., Ding W., Zhang J., Li Y., Luo J., Bolan N., Xie Z. (2014). Biochar suppressed the decomposition of organic carbon in a cultivated sandy loam soil: A negative priming effect. Soil Biol. Biochem..

[57] Freddo A., Cai B.J., Reid V. (2012). Environmental contextualisation of potential toxic elements and polycyclic aromatic hydrocarbons in biochar. Environ. Pollut..

[58] Ali L., Palamanit A., Techato K., Ullah A., Chowdhury M.S., Phoungthong K. (2022). Characteristics of Biochars Derived from the Pyrolysis and Co-Pyrolysis of Rubberwood Sawdust and Sewage Sludge for Further Applications. Sustainability.

[59] Singh B., Dolk M.M., Shen Q., Camps-Arbestain M., Singh B., Camps-Arbestain M., Lehmann J. (2017). Biochar pH, electrical conductivity and liming potential. Biochar: A Guide to Analytical Methods.

[60] Malińska K., Miełgieś K. (2016). Current quality and legal reguirements for biochar as a fertilizers and soil improver. Sci. Work. Inst. Ceram. Build. Mater..

[61] Malińska K. (2015). Legal and quality aspects of requirements defined for biochar. Inż. I Ochr. Sr..

[62] Pereira R.C., Muetzel S., Arbestain M.C., Bishop P., Hina K., Hedley M. (2014). Assessment of the influence of biochar on rumen and silage fermentation: A laboratory–scale experiment. Anim. Feed Sci. Technol..

[63] Tang J., Zhy W., Kookana R., Katayama A. (2013). Characteristics of biochar and its application in remediation of contaminated soil. J. Biosci. Bioeng..

[64] Mohan D., Sarswat A., Ok S.Y., Pittman C.U. (2014). Organic and inorganic contaminants removal from water with biochar, a renewable, low cost and sustainable adsorbent—A critical review. Bioresour. Technol..

[65] Steiner C., Das K.C., Melear N., Lakly D. (2010). Reducing nitrogen loss during poultry litter composting using biochar. J. Environ. Qual..

[66] Steiner C., Melear N., Harris K., Das K.C. (2011). Biochar as bulking agent for poultry litter composting. Carbon Manag..

[67] Malińska K., Zabochnicka-Świątek M., Dach J. (2014). Effects of biochar amendment on ammonia emission during composting of sewage sludge. Ecol. Eng..

[68] Malińska K., Dach J. (2015). Biochar as a supplementary material for biogas production. Ecol. Eng..

[69] Paethanom A., Bartocci P., D’ Alessandro B., D’Amico M., Testarmata F., Moriconi N., Slopiecka K., Yoshikawa K., Fantozzi F. (2013). A low-cost pyrogas cleaning system for power generation: Scaling up from lab to pilot. Appl. Energy.

[70] Bartocci P., Bidini G., Saputo P., Fantozzi F. (2016). Biochar pellet carbon footprint. Chem. Eng. Trans..

[71] Bartocci P., Zampilli M., Bidini G., Fantozzi F. (2018). Hydrogen-rich gas production through steam gasification of charcoal pellet. Appl. Therm. Eng..

[72] Schmidt H.-P., Hagemann N., Abächerli F., Leifeld J., Bucheli T. (2021). Protecting the Climate with Biochar. Agroscope Sci..

[73] Ma R., Levard C., Judy J.D., Unrine J.M., Durenkamp M., Martin B., Jefferson B., Lowry G.V. (2014). Fate of zinc oxide and silver nanoparticles in a pilot wastewater treatment plant and in processed biosolids. Environ. Sci. Technol..

[74] Malghani S., Gleixner G., Trumbore S.E. (2013). Chars produced by slow pyrolysis and hydrothermal carbonization vary in carbon sequestration potential and greenhouse gases emissions. Soil Biol. Biochem..

[75] Tan Z., Yuan S. (2019). The effect of preparing temperature and atmosphere on biochar’s quality for soil improving. Waste Biomass Valor..

[76] Tan X.F., Liu S.B., Liu Y.G., Gu Y.L., Zeng G.M., Hu X.J., Wang X., Liu S.H., Jiang L.H. (2017). Biochar as potential sustainable precursors for activated carbon production: Multiple applications in environmental protection and energy storage. Bioresour. Technol..

[77] El-Naggar A., Lee S.S., Rinklebe J., Farooq M., Song H., Sarmah A.K., Zimmerman A.R., Ahmad M., Shaheen S.M., Ok Y.S. (2019). Biochar application to low fertility soils: A review of current status, and future prospects. Geoderma.

[78] Nanda S., Dalai A.K., Berruti F., Kozinski J.A. (2016). Biochar as an exceptional bioresource for energy, agronomy, carbon sequestration, activated carbon and specialty materials. Waste Biomass Valor..

[79] Osman A.I., Fawzy S., Farghali M., El-Azazy M., Elgarahy A.M., Fahim R.A., Maksoud M.I.A.A., Ajlan A.A., Yousry M., Saleem Y. (2022). Biochar for agronomy, animal farming, anaerobic digestion, composting, water treatment, soil remediation, construction, energy storage, and carbon sequestration: A review. Environ. Chem. Lett..

[80] Hameed A., Hussain S.A., Yang J., Ijaz M.U., Liu Q., Suleria H.A.R., Song Y. (2017). Antioxidants potential of the filamentous fungi (*Mucor circinelloides*). Nutrients.

[81] Wu M., Feng Q., Sun X., Wang H., Gielen G., Wu W. (2015). Rice (*Oryza sativa* L.) plantation affects the stability of biochar in paddy soil. Sci. Rep..

[82] Chen B., Zhou D., Zhu L. (2008). Transitional adsorption and partition of nonpolar andpolar aromatic contaminants by biochar of pine needles with different pyrolytic temperatures. Environ. Sci. Technol..

[83] Zielińska A., Oleszczuk P. (2015). The conversion of sewage sludge into biocharreduces polycyclic aromatic hydrocarbon content and ecotoxicity butincreases trace metal content. Biomass Bioenergy.

[84] Oleszczuk P., Jośko I., Kuśmierz H. (2013). Biochar properties regarding to contaminants content and ecotoxicological assessment. J. Hazard. Mater..

[85] Kim J.H., Ok Y.S., Choi G.-H., Park B.-J. (2015). Residual perfluoro chemicals in the biochar from sewage sludge. Chemosphere.

[86] Spokas K. (2010). Review of the stability of biochar in soils: Predictability of O:C molar. Carbon Manag..

[87] Brassard P., Godbout S., Lévesque V., Palacios J.H., Raghavan V., Ahmed A., Hogue R., Jeanne T., Verma M. (2019). Biochar for Soil Amendment. Char and Carbon Materials Derived from Biomass.

[88] Yu J., Reina T.R., Paterson N., Millan M. (2022). On the primary pyrolysis products of torrefied oak at extremely high heating rates in a wire mesh reactor. Appl. Energy Combust. Sci..

[89] Rozkrut D., Statistics Poland (2021). Statistical Yearbook of Forestry.

[90] Tumuluru J.S., Boardman R.D., Wright C.T., Hess J.R. (2012). Some chemical compositional changes in miscanthus and white oak sawdust samples during torrefaction. Energies.

[91] Ohtsuka T., Tomotsune M., Ando M., Tsukimori Y., Koizumi H., Yoshitake S. (2021). Effects of the application of biochar to plant growth and net primary production in an oak forest. Forests.

[92] Saletnik B., Bajcar M., Saletnik A., Zaguła G., Puchalski C. (2021). Effect of the Pyrolysis Process Applied to Waste Branches Biomass from Fruit Trees on the Calorific Value of the Biochar and Dust Explosivity. Energies.

[93] Stelte W., Holm J., Sanadi A., Barsberg S., Ahrenfeldt J., Henriksen U. (2011). Fuel pellets from biomass: The importance of the pelletizing pressure and its dependency on the processing conditions. Fuel.

[94] EN14034, Part 2 (2011). Determination of Explosion Characteristics of Dust Clouds—Part 2: Determination of the Maximum Rate of Explosion Pressure Rise (dp/dt) Max of Dust Clouds. https://infostore.saiglobal.com/en-us/Standards/EN-14034-2-2006-A1-2011-328536_SAIG_CEN_CEN_756384.

[95] British Standards Insitution (2011). Solid Biofuels—Determination of Total Carbon, Hydrogen and Nitrogen Content—Instrumental Methods.

[96] British Standards Institution (2010). Solid Biofuels—Determination of Ash.

[97] British Standards Institution (2011). Solid Biofuels—Determination of Volatile Substances.

[98] British Standards Institution (2010). Solid Biofuels—Determination of Calorific Value.

[99] Qian X. (2019). Statistical Analysis and Evaluation of the Advanced Biomass and Natural Gas Co-Combustion Performance. Ph.D. Thesis.

[100] Chen W.H., Lin Y.Y., Liu H.C., Baroutian S. (2020). Optimization of food waste hydrothermal liquefaction by a two-step process in association with a double analysis. Energy.

[101] Kazimierski P., Januszewicz K., Godlewski W., Fijuk A., Suchocki T., Chaja P., Barczak B., Kardaś D. (2022). The Course and the Effects of Agricultural Biomass Pyrolysis in the Production of High-Calorific Biochar. Materials.

[102] Tomczyk A., Sokołowska Z., Boguta P. (2020). Biochar physicochemical properties: Pyrolysis temperature and feedstock kind effects. Rev. Environ. Sci. Biotechnol..

[103] Tag A.T., Duman G., Ucar S., Yanik J. (2016). Effects of feedstock type and pyrolysis temperature on potential applications of biochar. J. Anal. Appl. Pyrolysis.

[104] Cantrell K.B., Hunt P.G., Uchimiya M., Novak J.M., Ro K.S. (2012). Impact of pyrolysis temperature and manure source on physicochemical characteristics of biochar. Bioresour. Technol..

[105] Enders A., Hanley K., Whitman T., Joseph S., Lehmann J. (2012). Characterization of biochars to evaluate recalcitrance and agronomic performance. Bioresour Technol..

[106] Kazimierski P., Kardaś D. (2016). Effect of temperature on the balance of carbon, hydrogen and nitrogen in pyrolysis products. Inż. I Apar. Chem..

[107] Kratofil M., Zarzycki R., Kobyłecki R., Bis Z. (2014). Badania procesu toryfikacji biomasy. Energy Policy J..

[108] Saletnik B., Bajcar M., Zaguła G., Czernicka M., Puchalski C. (2016). Impact of the biomass pyrolysis parameters on the quality of biocarbon obtained from rape straw, rye straw and willow chips. Econtechmod. Int. Q. J..

[109] Saletnik A., Saletnik B., Puchalski C. (2021). Modification of Energy Parameters in Wood Pellets with the Use of Waste Cooking Oil. Energies.

[110] Tong S., Xiao L., Li X., Zhu X., Liu H., Luo G., Worasuwannarak N., Kerdsuwan S., Fungtammasan B., Yao H. (2018). A gas-pressurized torrefaction method for biomass wastes. Energy Convers. Manag..

[111] Enes T., Aranha J., Fonseca T., Lopes D., Alves A., Lousada J. (2019). Thermal Properties of Residual Agroforestry Biomass of Northern Portugal. Energies.

[112] Sellappah V., Uemura Y., Hassan S., Sulaiman M.H., Lam M.K. (2016). Torrefaction of Empty Fruit Bunch in the Presence of Combustion. Gas. Procedia Eng..

[113] Dhyani V., Bhaskar T. (2018). A comprehensive review on the pyrolysis of lignocellulosic biomass. Renew Energy.

[114] Charvet F., Silva F., Ruivo L., Tarelho L., Matos A., Figueiredo da Silva J., Neves D. (2021). Pyrolysis Characteristics of Undervalued Wood Varieties in the Portuguese Charcoal Sector. Energies.

[115] Domingues R.R., Trugilho P.F., Silva C.A., de Melo I.C.N.A., Melo L.C.A., Magriotis Z.M., Sánchez-Monedero M.A. (2017). Properties of biochar derived from wood and high-nutrient biomasses with the aim of agronomic and environmental benefits. PLoS ONE.

[116] Jin Y., Liang X., He M., Liu Y., Tian G., Shi J. (2016). Manure biochar influence upon soil properties, phosphorus distribution and phosphatase activites: A microcosm incubation study. Chemosphere.

[117] Karaosmanoglu F. (2020). Biobriquetting of rapeseed cake. Energy Sources.

[118] Kołodyńska D., Wnętrzak R., Leahy J.J., Kwapiński W., Hayes M.H.B., Hubicki Z. (2012). Kinetic and adsorptive characterization of biochar in metal ions removal. Chem Eng. J..

[119] Mohan D., Rajput S., Singh V.K., Steele P.H., Pittman C.U. (2011). Modeling and evaluation of chromium remediation from water using low cost bio-char.; a green adsorbent. J. Hazard. Mater..

[120] Mullen C.A., Boateng A.A., Goldberg N.M., Lima I.M., Laird D.A., Hicks K.B. (2010). Bio-oil and bio-char production from corn cobs and stover by pyrolysis. Biomass Bioenergy.

[121] Novak J., Sigua G., Watts D., Cantrell K., Shumaker P., Szogi A., Johnson M.G., Spokas K. (2016). Biochars impact on water infiltration and water quality through a compacted subsoil layer. Chemosphere.

[122] Rafiq M.K., Bachmann R.T., Rafiq M.T., Shang Z., Joseph S., Long R. (2016). Influence of pyrolysis temperature on physico- chemical properties of corn stover (*Zea mays* L.) biochar and feasibility for carbon capture and energy balance. PLoS ONE.

[123] Uras U., Carrier M., Hardie A.G., Knoetze J.H. (2012). Physico-chemical characterization of biochars from vacuum pyrolysis of South African agricultural wastes for application as soil amendments. J. Anal. Appl. Pyrol..

[124] Venegas A., Rigol A., Vidal M. (2015). Viability of organic wastes and biochars as amendments for the remediation of heavy metal-contaminated soils. Chemosphere.

[125] Yao Y., Gao B., Zhang M., Inyang M., Zimmerman A.R. (2012). Effect of biochar amendment on sorption and leaching of nitrate, ammonium and phosphate in a sandy soil. Chemosphere.

[126] Zama E.F., Zhu Y.-G., Reid B.J., Sun G.-X. (2017). The role of biochar properties in influencing the sorption and desorption of Pb(II), Cd(II) and As(III) in aqueous solution. J. Clean. Prod..

[127] Bruckman V.J., Yan S., Hochbichler E., Glatzel G., Matovic M.D. (2013). Considerations for sustainable biomass production in quercusdominated forest ecosystems. Biomass Now-Cultivation and Utilization.

[128] Naeem M.A., Khalid M., Ahmad Z., Naveed M. (2016). Low pyrolysis temperature biochar improves growth and nutrient availability of Maize on Typic Calciargid. Commun. Soil Sci. Plant Anal..

[129] Fidel R.B., Laird D.A., Parkin T.B. (2017). Impact of biochar organic and inorganic carbon on soil CO_2_ and N_2_O emissions. J. Environ. Qual..

[130] Deng S., Tan H., Wang X., Yang F., Cao R., Wang Z., Ruan R. (2017). Investigation on the fast co-pyrolysis of sewage sludge with biomass and the combustion reactivity of residual char. Bioresour. Technol..

[131] Cao X., Ma L., Gao B., Harris W. (2009). Dairy-Manure Derived Biochar Effectively Sorbs Lead and Atrazine Environ. Sci. Technol..

[132] Szweykowska A. (1999). Plant Physiology.

[133] Wińska-Krysiak M. (2006). Calcium transporting proteins in plants. Acta Agrophysica.

[134] Bezak-Mazur E., Stoińska R. (2013). The importance of phosphorus in the environment—Review article. Arch. Waste Manag. Environ. Prot..

[135] Krzywy E. (2000). Fertilization of Soils and Plants.

[136] Xiao X., Chen B., Chen Z., Zhu L., Schnoor J.L. (2018). Insight into multiple and multilevel structures of biochars and their potential environmental applications: A critical review. Environ. Sci. Technol..

[137] Hossain M.K., Strezov V., Chan K.Y., Ziolkowski A., Nelson P.F. (2011). Influence of pyrolysis temperature on production and nutrient properties of wastewater sludge biochar. J. Environ. Manag..

[138] Jin H., Arazo R.O., Gao J., Capared S., Chang Z. (2014). Leaching of heavy metals from fast pyrolysis residues produced from different particle sizes of sewage sludge. J. Anal. Appl. Pyrol..

[139] Agrafiotia E., Bourasa G., Kalderisb D., Diamadopoulos E. (2013). Biochar production by sewage sludge pyrolysis. J. Anal. Appl. Pyrol..

[140] Lu T., Yuan H., Wang Y., Huang H., Chen Y. (2016). Characteristic of heavy metals in biochar derived from sewage sludge. J. Mater. Cycles Waste Manag..

[141] Çengel Y.A., Boles M.A. (2006). Thermodynamics: An Engineering Approach.

[142] Slatter D.J.F., Sattar H., Medina C.H., Andrews G.E., Phylaktou H.N., Gibbs B.M. (2015). Biomass explosion testing: Accounting for the post-test residue and implications on the results. J. Loss Prev. Proc..

[143] Bajcar M., Saletnik B., Zaguła G., Puchalski C. (2020). Analysis of the Effect of the Biomass Torrefaction Process on Selected Parameters of Dust Explosivity. Molecules.

[144] Saletnik B., Saletnik A., Zaguła G., Bajcar M., Puchalski C. (2022). The Use of Wood Pellets in the Production of High Quality Biocarbon Materials. Materials.

[145] Cashdollar K.L. (2000). Overview of dust explosibility characteristics. J. Loss Prev. Process Ind..

[146] Cordero T., Marquez F., Rodriquez-Mirasol J., Rodriguez J.J. (2001). Predicting heating values of lignocellulosic and carbonaceous materials from proximate analysis. Fuel.

[147] Demirbaş A. (1997). Calculation of higher heating values of biomass fuels. Fuel.

